# Characterization of Non-Food Foreign Bodies Aspirated by Children: A Systematic Review of the Literature

**DOI:** 10.3390/children10101709

**Published:** 2023-10-20

**Authors:** Giulia Lorenzoni, Marco Vertuani, Valeria Basso, Paola Rescigno, Honoria Ocagli, Dario Gregori

**Affiliations:** Unit of Biostatistics, Epidemiology and Public Health, Department of Cardiac, Thoracic, Vascular Sciences, and Public Health, University of Padova, 35131 Padova, Italy; giulia.lorenzoni@unipd.it (G.L.); marco.vertuani@ubep.unipd.it (M.V.); valeria.basso@ubep.unipd.it (V.B.); paola.rescigno@ubep.unipd.it (P.R.); honoria.ocagli@unipd.it (H.O.)

**Keywords:** foreign body, aspiration, children, button batteries

## Abstract

Background. Foreign Body Aspiration (FBA) represents a leading cause of death among unintentional injuries in children less than one year of age. This study reviewed case reports and case series reporting non-food FBA in children to characterize aspirated foreign bodies, describing the clinical presentations and the outcomes. Methods: A systematic review was conducted according to the PRISMA checklist. Case reports and case series presenting non-food FBA in children (up to 18 years) were eligible to be included. Information regarding study characteristics, child demographics, foreign body characteristics, clinical presentation, and outcome were extracted. Results: The review included 248 articles published between 1965 and 2023, corresponding to 294 cases. The male gender was the most prevalent (194 cases, 66%), and the median age was 3.5 years (Interquartile Range: 1–8 years). Button batteries were the objects most frequently reported (21 cases, 7.1%). Objects were located most often in the bronchus (102 cases, 35%). The most common symptom was cough (181 cases, 62%), followed by respiratory distress (160 cases, 54%) and wheezing/stridor (127 cases, 43%). Conclusions: The present systematic review may have relevant public health implications, since characterizing objects that cause foreign body injuries is essential to reduce the burden of this phenomenon.

## 1. Introduction

Foreign Body Aspiration (FBA) represents a leading cause of death among unintentional injuries in children under one year of age [1]. It continues to represent a relevant public health problem until age 14. Children are at high risk for aspiration due to a wide range of psycho-physiological factors. They have incomplete dentition, poor swallowing coordination, and small-diameter airways, making them at greater risk of complete airway obstruction than older children [1]. Regarding behavioral aspects, mouthing activity, inability to distinguish edible from non-edible objects, and easy distractibility (e.g., eating and playing simultaneously) are the factors that most expose them to a high risk of aspiration. Historically, FBAs were considered “accidents” that occurred by chance. However, growing knowledge of the phenomenon has highlighted that they are predictable and preventable events and are referred to as “injuries” rather than “accidents” [2,3].

The first and most important tool for preventing FBAs is epidemiological surveillance of the phenomenon, based on which specific primary prevention measures could be developed [4]. The case of toys represents a clear example of the fact that these injuries are preventable phenomena through ad hoc prevention actions based on epidemiological surveillance. Toys were a leading cause of foreign body injuries for a long time. However, the introduction of ad hoc legislation to increase the safety of toys and nursery products and the development of public health campaigns aimed at raising awareness of the phenomenon resulted in a reduction in the burden of foreign body injuries caused by these products [5].

Unfortunately, epidemiological surveillance of FBA can be challenging. There is no specific mandatory reporting of foreign body injury cases, and only the most severe cases (or those requiring medical intervention to remove the foreign body) come to the attention of health care providers, with a resulting hidden burden of injuries self-resolved at home [6].

However, the most severe cases that come to the attention of healthcare providers, even though not exhaustive, also can contribute to the epidemiological surveillance of this phenomenon. First, they allow for characterizing the items that have resulted in the most severe injuries, contributing to identifying emerging hazards that can be reported promptly to the health authorities. A classic example is that of gel candies containing konjac [7]. In the United States, in the early 2000s, a considerable number of deaths and near-deaths were observed in a relatively short time due to the aspiration of these candies [8]. Following these reports, the Food and Drug Administration (FDA) promptly introduced a series of actions to prevent new episodes of choking on these candies by declaring them “unfit for food”.

In addition, sharing and studying cases that come to the attention of healthcare providers can help increase awareness and expertise within the scientific community to diagnose and manage this type of injury. In fact, recognizing and managing this type of injury is not always straightforward [9] and often relies on the experience of the clinician/hospital. The clinical presentation of FBA cases is widely heterogeneous and depends on the type of foreign body, anatomic location, degree of obstruction, complications, and time since aspiration. Cautious evaluation combining clinical examination with careful questioning regarding the possibility of aspiration is essential to prevent missed cases, especially when non-specific symptoms such as cough and fever are present [10]. A recent study of a series of hospital-assessed cases of FBA showed that about one-third were misdiagnosed, and about half were misdiagnosed with bronchiolitis [11]. Children with no specific symptoms presenting with unwitnessed FBA for more than 24 h and involving food (generally radiolucent) were more likely to receive a misdiagnosis [11]. This study systematically reviewed case reports and case series reporting non-food FBA in children to characterize aspirated foreign bodies, describing the clinical presentations and outcomes.

## 2. Materials and Methods

### 2.1. Protocol and Registration

A systematic review was conducted according to the PRISMA checklist [12] (Table S1, Supplementary Materials) and registered in the PROSPERO online database (PROSPERO identifier: CRD42023463546).

### 2.2. Search Strategy and Information Sources

A search strategy was created combining the concepts related to foreign bodies, airway obstruction, child, and case reports or case series. The search was done on PubMed and Scopus without language and time limitations. Table S2 reports the search strategy.

### 2.3. Eligibility Criteria

The following inclusion criteria were considered: pediatric age group (up to 18 years), involvement of the airways (nose, pharynx, larynx, bronchi, and lungs), and presence of a non-food foreign body description in the article, case reports, or case series. The exclusion criteria were the following: food foreign body, different location (digestive tract, orbits, ears, mouth, brain, joints, and genitalia), use of weapons, blunt force trauma, abuse, adult subjects (older than 18 years), penetrating foreign body, differentiation of respiratory disease from foreign body (in the absence of a foreign body), absence of a foreign body description in the article, different study design than a case report or case study (e.g., reviews, systematic reviews, meta-analysis, and randomized controlled trials), non-English study, and unavailability of the full-text.

### 2.4. Study Selection

An online systematic review software was used (Covidence systematic review software, Veritas Health Innovation, Melbourne, Australia). Two reviewers (M.V. and V.B.) independently screened references at first in the title/abstract screening and then in the full-text screening. Disagreements were solved with the help of a third expert (G.L. or H.O.). In the full-text screening phase, articles not meeting the inclusion criteria or those that were unavailable were excluded.

### 2.5. Data Extraction

The data were independently extracted from articles by at least two independent authors (M.V., V.B. and P.R.). The following information was extracted: (1) characteristics of the study (year of publication, authors, case series/case report, and country), (2) characteristics of the subject (gender and age), (3) foreign body characteristics (type, location, shape, length, height, diameter, and color), (4) clinical presentation (choking, cough, wheeze and stridor, respiratory distress, fever, cyanosis, intense pain, nausea and vomit, and cardiorespiratory arrest), (5) outcome (death or complications), (6) methods employed for foreign body removal (surgery/endoscopy/per via naturalis).

### 2.6. Quality of Studies

The Joanna Briggs Institute’s critical appraisal tools were used to evaluate the methodological quality of a study for case reports [13] and case series [14]. In both the checklists, the question was rated as ‘yes’, ‘no’, ‘unclear’, or ‘not applicable’. Three independent reviewers assessed the methodological quality of the included articles (V.B., P.R. and M.V.). The case report checklist consisted of 8 questions, whereas the case series had 10 questions. Consensus was reached through discussion.

### 2.7. Statistical Analyses

Descriptive statistics were reported as the median and Interquartile Range (IQR) for continuous variables and as percentages (absolute numbers) for categorical variables.

Variable distribution was compared between subjects of one year of age and less (infants) and subjects over the age of one, because infants are well known to be at the highest risk for FBA and for death from FBA The Wilcoxon test was employed to compare the distribution of continuous variables. The Chi-squared test and Fisher’s exact test (whichever was appropriate) were employed to compare the distribution of categorical variables.

The changes in the number of case reports and case series published over the years were evaluated using a regression approach.

Analyses were performed using the R software 4.2.2.

## 3. Results

**Figure 1:**
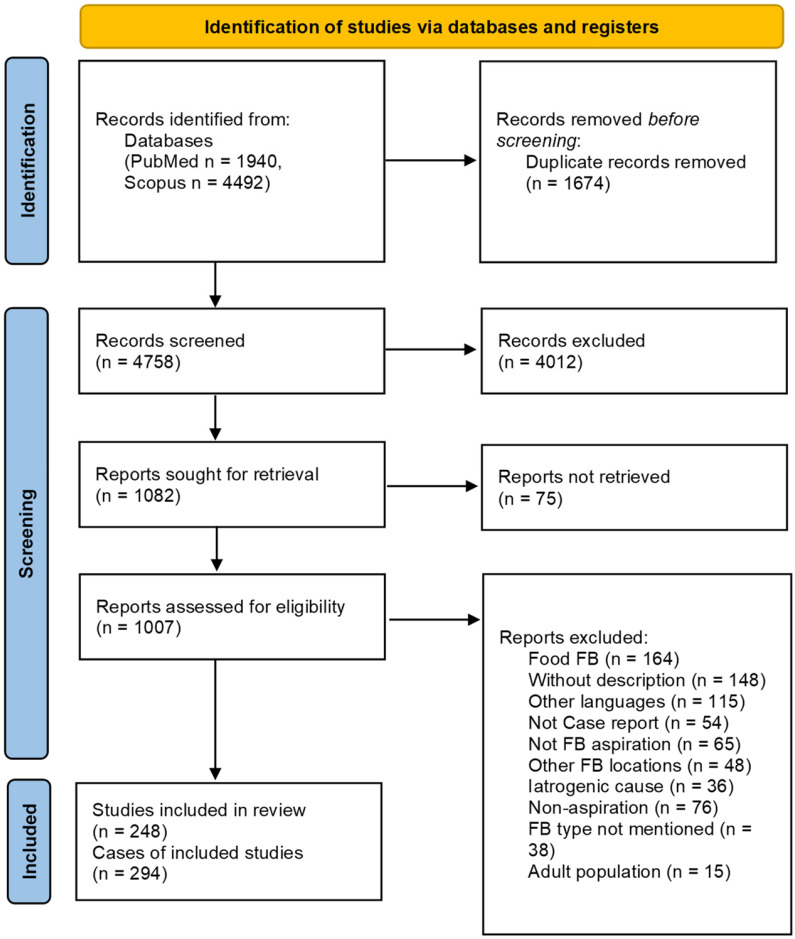
PRISMA flow diagram of the included studies.

The systematic review included 248 articles published between 1965 and 2023, corresponding to 294 cases. The PRISMA flowchart is presented in Figure 1: 4758 articles were screened in the title/abstract phase, and 4012 were considered ineligible; in the full-text phase, 1082 records were considered, and the full-text of 75 of these were unavailable, so 1007 articles were screened.

**Figure 2:**
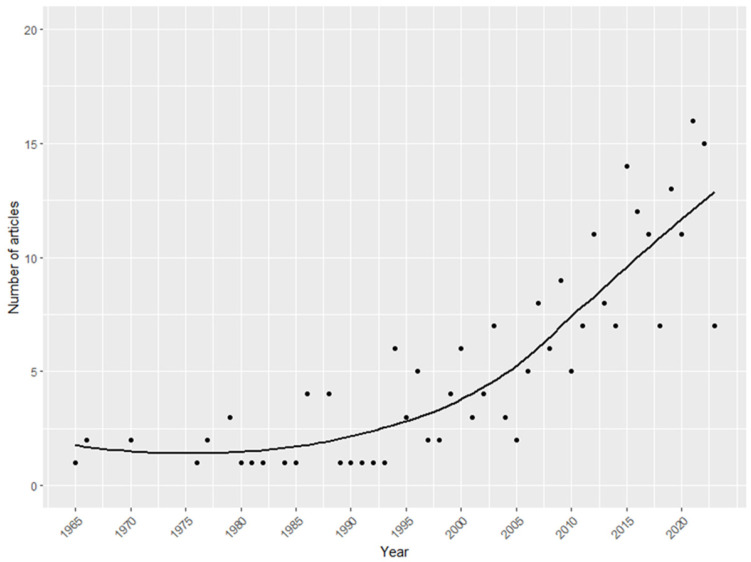
Number of case reports and case series published per year. The time series were fitted using local polynomial regression. The dots represent the observed data.

Table S3 (Supplementary Materials) presents the full list of cases included, while Table S4 presents the list of references. The number of case reports and case series of FBA increased significantly (*p*-value < 0.001) over the years (Figure 2).

Table 1 presents the FBA cases’ characteristics. The male gender was the most prevalent (194 cases, 66%), and the median age was 3.5 years (IQR: 1–8 years). In almost half of the cases (43%), the report was from an Asian country, mainly India (52 cases); the remaining were from Europe (57, 19%), America (90, 31%), Africa (14, 4.8%), and Australia (7, 2.4%).

### 3.1. Foreign Body Characteristics

The complete list of aspirated foreign bodies is presented in Table S5 (Supplementary Materials). Button batteries were the objects most frequently reported (21 cases, 7.1%), followed by toys (20 cases, 6.8%). Button batteries were found most often in the nose. Objects most often had a sharp shape (57 cases, 19%), followed by a round shape (48 cases, 16%) and irregular shape (45 cases, 15%). The median diameter and width were less than 1 cm (0.8 cm), while the median length was 2 cm (IQR: 1.4–3.2 cm). The colors of aspirated objects were mostly neutral, i.e., in 37% of cases, they were grey (Table 1).

### 3.2. Clinical Presentation and Outcomes

Objects were located most often in the bronchus (102 cases, 35%). Unfortunately, it was not possible to specify if the left or the right bronchus was involved because the information was not always reported. The most common symptom was cough (181 cases, 62%), followed by respiratory distress (160 cases, 54%) and wheezing/stridor (127 cases, 43%). Less than 10% of cases were asymptomatic. In 71 cases (24%), the FBA resulted in a complication. The most common complications were perforation/laceration and respiratory infection.

Most cases (199, 68%) underwent foreign body removal through endoscopy. However, in 88 cases (30%), surgical treatment was required for foreign body removal.

Thirteen cases resulted in death from FBA (4.4%) (see Table S3 for case characteristics). They were published between 1965 and 2022. All death cases involved an infant, except for one which involved a six-year-old. The male gender was the most prevalent (10 out of 13 cases). The foreign body location was most often the trachea (6 cases), larynx (3 cases), pharynx (2 cases), bronchus (1 case), and lungs (1 case). The foreign body type was heterogeneous, but the shape was most often round (4 cases) or sharp (4 cases).

### 3.3. FBA Cases According to Child Age

A total of 94 out of 294 cases (32%) involved an infant (Table 2). No significant differences were detected in gender distribution, confirming a higher proportion of males compared to females in both age classes (62% of males vs. 38% of females in infants and 68% of males and 32% of females in children above one year of age).

The analysis according to child age (infants vs. children over one year) showed a significant difference in the distribution of the foreign body location. Foreign bodies were most often located in the larynx of infants (36 cases, 38%), followed by the bronchus (22 cases, 23%), the pharynx (16 cases, 17%), the trachea (13 cases, 14%), and lungs (7 cases, 7.4%). Children over the age of one most often presented with foreign bodies in the bronchus (80 cases, 40%), followed by larynx (32 cases, 16%), and the nose (25 cases, 12%). Interestingly, all foreign bodies in the nose were identified in children over one.

No significant differences were identified in foreign body dimensions: the median of the foreign bodies’ width and diameter was less than 1 cm in both age classes, while the length was about 2 cm.

Conversely, a different clinical presentation was detected. Infants were more likely to present with wheezing/stridor (56 cases, 60%), respiratory distress (66 cases, 70%), and cyanosis (29 cases, 31%). Children over one were more likely to suffer from cough (120 cases, 60%).

### 3.4. Quality Assessment

Tables S6 and S7 report the quality assessment of the included studies. The quality evaluation of individual studies is described in detail in Table 3. Most of the case reports adequately described the patients’ demographic characteristics (227, 97%), the clinical presentation (229, 98%), the assessment methods (222, 95%), and the procedure/intervention (218, 93%), and most provided a takeaway lesson (219, 94%). The patient’s history (203, 87%), the post-intervention clinical condition (202, 86%), and the adverse events (184, 79%) were identified less clearly in the case report studies. In the case series, the questions clearly reported were those related to the inclusion criteria (13, 93%), the use of valid methods for identification of the condition for all participants included (13, 93%), and the complete inclusion of participants (14, 100%), whereas the condition measurement (12, 86%) and the consecutive inclusion criteria (12, 86%) were less clearly reported. The reporting of the demographics of the participants included in the study (10, 71%), the outcomes or follow-up (11, 79%), and the presenting sites’/clinics’ demographic information (10, 71%) were adequately identified. Only eight studies (57%) reported an appropriate statistical analysis.

## 4. Discussion

The present work systematically reviewed case reports and case series presenting FBA cases published in the literature. Interestingly, the number of publications increased significantly over the years, highlighting an increasing awareness of the researchers regarding the issue of foreign body injuries in children.

Subjects’ characteristics were consistent with previous studies on FBA. Infants and subjects of male gender were those most prone to FBA. Children under one year of age are well known to be at the highest risk for aspiration [15], while the male gender is most often associated with any type of injury, including foreign body ones [16]. Furthermore, even though only a small proportion of deaths was detected, all death cases except for one occurred in infants. This information reflects international data showing that aspiration is the leading cause of death among unintentional injuries in children under the age of one [17].

Interestingly, the analysis of foreign body characteristics showed that about two-thirds of the objects had an irregular and/or sharp shape. This was probably one of the main reasons for the high proportion of complications (24%), which in turn made the case reports more likely to be published. Conversely, the literature generally reports that round-shaped objects, such as marbles, are most frequently involved in foreign body injuries [18].

The study detected a wide variety of aspirated objects. It is noteworthy that button batteries continue to be frequently reported. Their aspiration, ingestion or insertion in a body cavity is very dangerous, as demonstrated by international literature [19]. Batteries produce an electric current that hydrolyzes body fluids, causing tissue damage as early as 2 h after ingestion/aspiration [20]. There are several ongoing initiatives to prevent battery injuries [21], such as designing packaging that makes it difficult for a child to open the battery compartment in items that use batteries. However, the fact that battery injuries were frequently reported in the present study should not be interpreted as a sign that public health campaigns are ineffective. We cannot rule out that since, generally, battery injuries determine the most severe complications, they are more likely to be published as case reports.

The analysis of the clinical presentation showed that the most frequent symptoms were cough and wheezing. These are common symptoms in children of several pediatric diseases, making the diagnosis of FBA potentially tricky if the FBA is unwitnessed. The lack of a specific clinical presentation of FBA is a well-known challenge of FBA management because it may result in delayed diagnosis [22]. Delayed diagnosis and treatment of FBA are known to be associated with a high risk of complications (e.g., pneumonia, lung abscess, and fistula) that may require surgical management. The delay in diagnosing and treating foreign body injury is particularly relevant for high-risk objects such as button batteries [23]. Button batteries may cause tissue damage within 2 h from aspiration/ingestion. The initial tissue damage may lead to tissue necrosis and ulceration, resulting in fistula formation or tissue perforation with potential systemic effects such as infection or organ damage. Prompt diagnosis and treatment are even more important in these cases, but they could be challenging because of heterogeneous clinical presentations, and even after a foreign body is detected through radiological exams, a button battery could be mistaken for a coin. The present results showed that clinical presentation was slightly different among infants, presenting most often with acute signs/symptoms (e.g., respiratory distress), thus highlighting the need to consider the possibility of FBA in the differential diagnosis.

### 4.1. Public Health Implications and Practical Recommendations

The present systematic review may have relevant public health implications.

Characterizing objects that cause foreign body injuries is essential to reduce the burden of this phenomenon. First, it allows the identification of the characteristics (shape, size, and texture) of objects that most frequently cause this type of injury and which factors determine the most severe complications. This information is crucial for the activation of public health campaigns for primary prevention of the phenomenon.

Small objects are already well known to pose a high risk of aspiration for young children. The present results confirmed that objects involved in FBA had a small diameter/width (less than 1 cm). However, another relevant piece of information identified by this review is that sharp objects were often involved in FBAs, and these data would explain the considerable proportion of complications in this sample.

The primary prevention of non-food FBA would benefit from the partnership between public health agencies and industry. The management of aspiration risk involves industrial design by modifying (while already in the design phase) the features that make objects potentially dangerous. Together with the industrial design, the packaging can also help reduce the burden of this phenomenon, for example, by placing warning messages on the packaging of hazardous items, as is already done for small objects.

However, it is not only industry that plays a crucial role in the primary prevention of foreign body injuries. Prevention also involves educating the population, especially families, since the literature has shown that they are often unaware of FBA risk [24]. It is essential to develop informative campaigns that teach families to recognize dangerous objects based on shape, size, and material characteristics. For example, concerning the prevention of choking on food, it has been suggested to educate families during prenatal classes. The same approach also may be employed for non-food aspiration prevention.

### 4.2. Study Limitations

The main study limitation is represented by the fact that the present review was based on case reports and case series. The usefulness of reviewing clinical case reports is a matter of concern [25]. Considering only published case reports and case series for characterizing aspirated objects does not allow for a comprehensive overview of the phenomenon and presents several limitations; e.g., lack of generalizability, publication bias, and the retrospective nature of the study [25]. However, these descriptive studies often provide insights into rare conditions or unusual clinical presentations, alerting other healthcare professionals and stimulating further investigation [25]. From this point of view, their review would help identify early warnings for hypothesis generation and educational purposes. Since there are no well-established clinical guidelines for diagnosing and managing foreign body injuries, case reports allow for sharing experience within foreign body management among the medical and scientific communities. Another study limitation is represented by the fact that the 25 cases of nose foreign bodies could be the result of both aspiration and insertion (and the studies did not always specify the mechanism). However, it has been decided to include them nonetheless, as the nose is an anatomical structure belonging to the airways.

## 5. Conclusions

The present systematic review characterized FBA cases published in the literature. Young male children were confirmed to be the most frequently involved in these injuries. Despite the variety of foreign bodies aspirated, they share similar characteristics concerning the small width/diameter. Interestingly, different from previous studies, the foreign bodies were reported to have most often a sharp shape, probably because only the most severe/challenging/unusual FBA cases were published. This is perhaps why about a quarter of the cases resulted in a complication, mainly perforation. The clinical presentation was often not specific. The present results could be helpful in improving the management of injured children, highlighting the need to consider the possibility of FBA in the differential diagnosis of children presenting with respiratory symptoms, since FBA could result in severe complications if not adequately treated promptly.

## Figures and Tables

**Table 1:** Characteristics of the FBA cases. Data are the median (I quartile–III quartile) for continuous variables and absolute numbers (percentages) for categorical variables.

Characteristics	N	N = 294
Gender	294	
F		100 (34%)
M		194 (66%)
Age	294	3.5 (1.0, 8.0)
Country	294	
Africa		14 (4.8%)
America		90 (31%)
Asia		126 (43%)
Australia		7 (2.4%)
Europe		57 (19%)
Foreign body characteristics		
Shape	294	
Round		48 (16%)
Cone		13 (4.4%)
Cylindrical		40 (14%)
Elongated		40 (14%)
Irregular		45 (15%)
Oval		12 (4.1%)
Polygon		13 (4.4%)
Sharp		57 (19%)
Spherical		19 (6.5%)
Spiral		7 (2.4%)
Length	141	2.00 (1.40, 3.20)
Width	68	0.80 (0.50, 1.00)
Diameter	59	0.80 (0.50, 1.05)
Color	254	
Black		24 (9.4%)
Blue		12 (4.7%)
Brown		18 (7.1%)
Gold		4 (1.6%)
Green		16 (6.3%)
Grey		95 (37%)
Multicolor		6 (2.4%)
Orange		2 (0.8%)
Pink		5 (2.0%)
Red		15 (5.9%)
Transparent		22 (8.7%)
White		21 (8.3%)
Yellow		14 (5.5%)
Clinical presentation		
Location	294	
Bronchus		102 (35%)
Larynx		68 (23%)
Lungs		27 (9.2%)
Nasal cavity		25 (8.5%)
Pharynx		35 (12%)
Trachea		37 (13%)
Choking	294	
No		217 (74%)
Yes		77 (26%)
Cough	294	
No		113 (38%)
Yes		181 (62%)
Wheezing/stridor	294	
No		167 (57%)
Yes		127 (43%)
Respiratory distress	294	
No		134 (46%)
Yes		160 (54%)
Fever	294	
No		238 (81%)
Yes		56 (19%)
Cyanosis	294	
No		230 (78%)
Yes		64 (22%)
Pain	294	
No		220 (75%)
Yes		74 (25%)
Vomit	294	
No		269 (91%)
Yes		25 (8.5%)
Cardiorespiratory arrest	294	
No		286 (97%)
Yes		8 (2.7%)
Asymptomatic	294	
No		265 (90%)
Yes		29 (9.9%)
Outcomes		
Death	294	
No		281 (96%)
Yes		13 (4.4%)
Complications	294	
No		223 (76%)
Yes		71 (24%)

**Table 2:** Characteristics of the FBA cases according to child’s age. Data are the median (I quartile–III quartile) for continuous variables and absolute numbers (percentages) for categorical variables.

Characteristic	N	≤1, N = 94	>1, N = 200	*p*-Value
Gender	294			0.3
F		36 (38%)	64 (32%)	
M		58 (62%)	136 (68%)	
Country	294			0.5
Africa		2 (2.1%)	12 (6.0%)	
America		33 (35%)	57 (28%)	
Asia		37 (39%)	89 (44%)	
Australia		2 (2.1%)	5 (2.5%)	
Europe		20 (21%)	37 (18%)	
*Foreign body characteristics*				
Length	141	2.00 (1.30, 3.65)	2.10 (1.45, 3.00)	0.4
Width	68	0.90 (0.50, 1.20)	0.70 (0.50, 1.00)	0.5
Diameter	59	0.60 (0.48, 1.20)	0.90 (0.50, 1.05)	0.6
*Clinical presentation*				
Location	294			<0.001
Bronchus		22 (23%)	80 (40%)	
Larynx		36 (38%)	32 (16%)	
Lungs		7 (7.4%)	20 (10%)	
Nose		0 (0%)	25 (12%)	
Pharynx		16 (17%)	19 (9.5%)	
Trachea		13 (14%)	24 (12%)	
Choking	294			<0.001
No		57 (61%)	160 (80%)	
Yes		37 (39%)	40 (20%)	
Cough	294			0.4
No		33 (35%)	80 (40%)	
Yes		61 (65%)	120 (60%)	
Wheezing/stridor	294			<0.001
No		38 (40%)	129 (64%)	
Yes		56 (60%)	71 (36%)	
Respiratory distress	294			<0.001
No		28 (30%)	106 (53%)	
Yes		66 (70%)	94 (47%)	
Fever	294			0.7
No		75 (80%)	163 (82%)	
Yes		19 (20%)	37 (18%)	
Cyanosis	294			0.01
No		65 (69%)	165 (82%)	
Yes		29 (31%)	35 (18%)	
Pain	294			0.016
No		62 (66%)	158 (79%)	
Yes		32 (34%)	42 (21%)	
Vomit	294			0.2
No		83 (88%)	186 (93%)	
Yes		11 (12%)	14 (7.0%)	
Cardiorespiratory arrest	294			0.002
No		87 (93%)	199 (100%)	
Yes		7 (7.4%)	1 (0.5%)	
Asymptomatic	294			0.2
No		88 (94%)	177 (88%)	
Yes		6 (6.4%)	23 (12%)	
*Outcomes*				
Death	294			<0.001
No		82 (87%)	199 (100%)	
Yes		12 (13%)	1 (0.5%)	
Complications	294			0.12
No		66 (70%)	157 (78%)	
Yes		28 (30%)	43 (22%)	

**Table 3:** Risk of bias results of the included studies.

ROB Case Reports (N 234)	
Were patient’s demographic characteristics clearly described?	
No	7 (3.0%)
Yes	227 (97%)
Was the patient’s history clearly described and presented as a timeline?	
No	31 (13%)
Yes	203 (87%)
Was the current clinical condition of the patient on presentation described in detail?	
No	5 (2.1%)
Yes	229 (98%)
Were diagnostics tests or assessment methods and the results clearly described?	
No	12 (5.1%)
Yes	222 (95%)
Was the intervention(s) or treatment procedure(s) clearly described?	
No	16 (6.8%)
Yes	218 (93%)
Was the post-intervention clinical condition clearly described?	
No	32 (14%)
Yes	202 (86%)
Were adverse events (harms) or unanticipated events identified and described?	
No	50 (21%)
Yes	184 (79%)
Does the case report provide takeaway lessons?	
No	15 (6.4%)
Yes	219 (94%)
**ROB Case series (N 14)**	
Were there clear criteria for inclusion in the case series?	
No	1 (7.1%)
Yes	13 (93%)
Was the condition measured in a standard, reliable way for all participants included in the case series?	
No	2 (14%)
Yes	12 (86%)
Were valid methods used for identification of the condition for all participants included in the case series?	
No	1 (7.1%)
Yes	13 (93%)
Did the case series have consecutive inclusion of participants?	
No	2 (14%)
Yes	12 (86%)
Did the case series have complete inclusion of participants?	
Yes	14 (100%)
Was there clear reporting of the demographics of the participants included in the study?	
No	4 (29%)
Yes	10 (71%)
Was there clear reporting of clinical information of the participants?	
No	1 (7.1%)
Yes	13 (93%)
Were the outcomes or follow-up results of cases clearly reported?	
No	3 (21%)
Yes	11 (79%)
Was there clear reporting of the presenting sites’/clinics’ demographic information?	
No	4 (29%)
Yes	10 (71%)
Was statistical analysis appropriate?	
No	6 (43%)
Yes	8 (57%)

## Data Availability

The data are contained within the article or Supplementary Materials.

## Supplementary Materials

The following supporting information can be downloaded at: https://www.mdpi.com/article/10.3390/children10101709/s1, Table S1. PRISMA checklist, Table S2. Search strategy, Table S3. List and characteristics of cases included in the review, Table S4. List of references of the articles included in the review, Table S5. List of aspirated foreign bodies, Table S6. Joanna Briggs Institute (JBI) checklist score of the case reports included in the review, Table S7. Joanna Briggs Institute (JBI) checklist score of the case series included in the review.
