# Supplementary material for: Characterization of Non-Food Foreign Bodies Aspirated by Children: A Systematic Review of the Literature
Source: Children (Basel). 2023 Oct 20;10(10):1709. doi: 10.3390/children10101709 (PMC10605452; doi:10.3390/children10101709)
Supplement: Supplementary file 1 [file children-10-01709-s001.zip › children-2667262-supplementary.pdf]

Supplementary Material

Table S1. PRISMA checklist

Section and Topic	Item #	Checklist item	Location where item is reported
**TITLE**			
Title	1	Identify the report as a systematic review.	1
**ABSTRACT**			
Abstract	2	See the PRISMA 2020 for Abstracts checklist.	1
**INTRODUCTION**			
Rationale	3	Describe the rationale for the review in the context of existing knowledge.	1-2
Objectives	4	Provide an explicit statement of the objective(s) or question(s) the review addresses.	1-2
**METHODS**			
Eligibility criteria	5	Specify the inclusion and exclusion criteria for the review and how studies were grouped for the syntheses.	2-3
Information sources	6	Specify all databases, registers, websites, organisations, reference lists and other sources searched or consulted to identify studies. Specify the date when each source was last searched or consulted.	2-3
Search strategy	7	Present the full search strategies for all databases, registers and websites, including any filters and limits used.	2-3
Selection process	8	Specify the methods used to decide whether a study met the inclusion criteria of the review, including how many reviewers screened each record and each report retrieved, whether they worked independently, and if applicable, details of automation tools used in the process.	2-3
Data collection process	9	Specify the methods used to collect data from reports, including how many reviewers collected data from each report, whether they worked independently, any processes for obtaining or confirming data from study investigators, and if applicable, details of automation tools used in the process.	2-3
Data items	10a	List and define all outcomes for which data were sought. Specify whether all results that were compatible with each outcome domain in each study were sought (e.g. for all measures, time points, analyses), and if not, the methods used to decide which results to collect.	2-3
	10b	List and define all other variables for which data were sought (e.g. participant and intervention characteristics, funding sources). Describe any assumptions made about any missing or unclear information.	2-3
Study risk of bias assessment	11	Specify the methods used to assess risk of bias in the included studies, including details of the tool(s) used, how many reviewers assessed each study and whether they worked independently, and if applicable, details of automation tools used in the process.	3
Effect measures	12	Specify for each outcome the effect measure(s) (e.g. risk ratio, mean difference) used in the synthesis or presentation of results.	NA
Synthesis methods	13a	Describe the processes used to decide which studies were eligible for each synthesis (e.g. tabulating the study intervention characteristics and comparing against the planned groups for each synthesis (item #5)).	2-3
	13b	Describe any methods required to prepare the data for presentation or synthesis, such as handling of missing summary statistics, or data conversions.	2-3

Section and Topic	Item #	Checklist item	Location where item is reported
	13c	Describe any methods used to tabulate or visually display results of individual studies and syntheses.	2-3
	13d	Describe any methods used to synthesize results and provide a rationale for the choice(s). If meta-analysis was performed, describe the model(s), method(s) to identify the presence and extent of statistical heterogeneity, and software package(s) used.	3
	13e	Describe any methods used to explore possible causes of heterogeneity among study results (e.g. subgroup analysis, meta-regression).	NA
	13f	Describe any sensitivity analyses conducted to assess robustness of the synthesized results.	NA
Reporting bias assessment	14	Describe any methods used to assess risk of bias due to missing results in a synthesis (arising from reporting biases).	3
Certainty assessment	15	Describe any methods used to assess certainty (or confidence) in the body of evidence for an outcome.	NA
**RESULTS**			
Study selection	16a	Describe the results of the search and selection process, from the number of records identified in the search to the number of studies included in the review, ideally using a flow diagram.	3-4
	16b	Cite studies that might appear to meet the inclusion criteria, but which were excluded, and explain why they were excluded.	NA
Study characteristics	17	Cite each included study and present its characteristics.	4-5
Risk of bias in studies	18	Present assessments of risk of bias for each included study.	9
Results of individual studies	19	For all outcomes, present, for each study: (a) summary statistics for each group (where appropriate) and (b) an effect estimate and its precision (e.g. confidence/credible interval), ideally using structured tables or plots.	5-9
Results of syntheses	20a	For each synthesis, briefly summarise the characteristics and risk of bias among contributing studies.	NA
	20b	Present results of all statistical syntheses conducted. If meta-analysis was done, present for each the summary estimate and its precision (e.g. confidence/credible interval) and measures of statistical heterogeneity. If comparing groups, describe the direction of the effect.	NA
	20c	Present results of all investigations of possible causes of heterogeneity among study results.	NA
	20d	Present results of all sensitivity analyses conducted to assess the robustness of the synthesized results.	NA
Reporting biases	21	Present assessments of risk of bias due to missing results (arising from reporting biases) for each synthesis assessed.	NA
Certainty of evidence	22	Present assessments of certainty (or confidence) in the body of evidence for each outcome assessed.	NA
**DISCUSSION**			
Discussion	23a	Provide a general interpretation of the results in the context of other evidence.	10-13
	23b	Discuss any limitations of the evidence included in the review.	10-13
	23c	Discuss any limitations of the review processes used.	10-13
	23d	Discuss implications of the results for practice, policy, and future research.	10-13
**OTHER INFORMATION**			

Section and Topic	Item #	Checklist item	Location where item is reported
Registration and protocol	24a	Provide registration information for the review, including register name and registration number, or state that the review was not registered.	2
	24b	Indicate where the review protocol can be accessed, or state that a protocol was not prepared.	NA
	24c	Describe and explain any amendments to information provided at registration or in the protocol.	NA
Support	25	Describe sources of financial or non-financial support for the review, and the role of the funders or sponsors in the review.	NA
Competing interests	26	Declare any competing interests of review authors.	NA
Availability of data, code and other materials	27	Report which of the following are publicly available and where they can be found: template data collection forms; data extracted from included studies; data used for all analyses; analytic code; any other materials used in the review.	NA

Table S2. Search strategy

Last update: 13/09/2023	
Time limits: None	
PubMed	("Foreign Bodies"[Mesh] OR "Foreign Bodies"[Title/Abstract] OR "Foreign Body"[Title/Abstract] OR “Foreign Objects”[Title/Abstract] OR “Foreign Object”[Title/Abstract])<br>AND<br>("Airway Obstruction"[Mesh] OR "Airway Obstruction"[Title/Abstract] OR “Airway Obstructions”[Title/Abstract] OR “Airway”[Title/Abstract] OR “Choking”[Title/Abstract] OR “Inhaled”[Title/Abstract] OR “Aspiration”[Title/Abstract] OR “Injury”[Title/Abstract] OR “Injuries”[Title/Abstract] OR “Pharynx”[Title/Abstract] OR “Larynx”[Title/Abstract] OR “Trachea”[Title/Abstract] OR “Bronchus”[Title/Abstract] OR “Lung”[Title/Abstract] OR “Lungs”[Title/Abstract])<br>AND<br>("Child"[Mesh] OR "Child"[Title/Abstract] OR “Children”[Title/Abstract] OR “Preschool Child”[Title/Abstract] OR “Preschool Children”[Title/Abstract] OR "Infant"[Mesh] OR "Infant"[Title/Abstract] OR “Infants”[Title/Abstract] OR “Newborns”[Title/Abstract] OR “Newborn”[Title/Abstract] OR “Neonate”[Title/Abstract] OR “Neonates”[Title/Abstract] OR “Toddler”[Title/Abstract] OR “Toddlers”[Title/Abstract])<br>AND<br>("case reports" [All Fields] OR "case report" [All Fields] OR "case series" [All Fields] OR "patient case" [All Fields] OR "case study" [All Fields] OR "case studies" [All Fields])
Scopus	(TITLE-ABS-KEY("Foreign Bodies") OR TITLE-ABS-KEY("Foreign Body") OR TITLE-ABS-KEY("Foreign Objects") OR TITLE-ABS-KEY("Foreign Object"))<br>AND<br>(TITLE-ABS-KEY("Airway Obstruction") OR TITLE-ABS-KEY("Airway Obstructions") OR TITLE-ABS-KEY("Airway") OR TITLE-ABS-KEY("Choking")

OR TITLE-ABS-KEY("Inhaled") OR TITLE-ABS-KEY("Aspiration") OR TITLE ABS-KEY("Injury") OR TITLE-ABS-KEY("Injuries") OR TITLE-ABS KEY("Pharynx") OR TITLE-ABS-KEY("Larynx") OR TITLE-ABS KEY("Trachea") OR TITLE-ABS-KEY("Bronchus") OR TITLE-ABS KEY("Lung") OR TITLE-ABS-KEY("Lungs"))

AND

(TITLE-ABS-KEY("Child") OR TITLE-ABS-KEY("Children") OR TITLE-ABS KEY("Preschool Child") OR TITLE-ABS-KEY("Preschool Children") OR TITLE ABS-KEY("Infant") OR TITLE-ABS-KEY("Infants") OR TITLE-ABS KEY("Newborns") OR TITLE-ABS-KEY("Newborn") OR TITLE-ABS KEY("Neonate") OR TITLE-ABS-KEY("Neonates") OR TITLE-ABS KEY("Toddler") OR TITLE-ABS-KEY("Toddlers"))

AND

(ALL ("case reports") OR ALL("case report") OR ALL("case series") OR ALL("patient case") OR ALL("case study") OR ALL("case studies"))

Table S3. List and characteristics of cases included in the review

Study	Country	Gender	Age (years)	Foreign Body Type	Location	Shape	Length	Width	Diameter	Color	Death	Treatment
Abbas 2022	Pakistan	M	8	Magnets	Bronchus	Elongated	2.8			Black	No	Surgical
Abbas 2022	Pakistan	F	7	Magnets	Bronchus	Elongated	1			Black	No	Surgical
Abder-Rahman 2009	Jordan	F	0.9	Pencil	Trachea	Elongated	5		0.7		Yes	Surgical
Abraham 2020	Tanzania	M	3	Pin	Bronchus	Sharp				Grey	No	Naturalis
Adoga 2009	Nigeria	M	13	Blade	Bronchus	polygon				Grey	No	Endoscopic
Ahad 1999	India	M	6	Wire	Trachea	Cylindrical	3	0.5			No	Endoscopic
Aihole 2019	India	M	12	Blade	Lungs	polygon				Grey	No	Endoscopic
Aihole 2020	India	F	0.8	Bead	Larynx	Spherical			0.3	Gold	No	Naturalis
Akhter 1994	USA	M	4	Toy	Bronchus	Irregular	0.7	0.5	0.2		No	Endoscopic
Al-Halfawy 2007	Egypt	F	16	Pin	Bronchus	Sharp	4	0.3		Grey	No	Endoscopic
Al-Halfawy 2007	Egypt	F	6	Safety pin	Trachea	Sharp	6	0.8		Grey	No	Endoscopic
Aljahdali 2021	Saudi Arabia	M	7	Can tab	Trachea	Conical	2	1.5		Grey	No	Endoscopic
AlKhalifah 2022	Saudi Arabia	F	0.8	Led	Bronchus	Elongated	2.1	0.5	0.5	Trasparent	No	Endoscopic
Alotaibi 2022	Saudi Arabia	F	7	Pen	Bronchus	Cylindrical	1.4		1	Blue	No	Endoscopic
Ambu 2001	Malaysia	M	3	Bird bones	Larynx	Elongated				White	No	Surgical
Anajar 2017	Morocco	F	6	Leech	Larynx	Oval				Black	No	Endoscopic
Anand 1979	India	M	3	Nail	Larynx	Sharp	1.5	1		Grey	No	Endoscopic
Antón-Pacheco 2008	Spain	M	5	Toothbrush cover	Trachea	Conical	4.5			White	No	Endoscopic
ArunBabu 2013	India	F	1.2	Sand	Bronchus	Irregular	0.4	0.3	0.2		No	Endoscopic
Arutyunyan 2014	USA	M	10	Pen	Bronchus	Cylindrical	1.3		0.5	Grey	No	Endoscopic
Asaf 2017	India	M	7	Magnets	Bronchus	Cylindrical	1.5			Black	No	Endoscopic
Atmaca 2009	Turkey	F	1	Plastic piece	Larynx	polygon	1.3	1.2		Red	No	Endoscopic
Atmaca 2011	Turkey	M	0.7	Seed (spiny cockleburs)	Larynx	Cylindrical	2			Yellow	No	Endoscopic
Azurara 2016	Portugal	M	10	Thumbtack	Bronchus	Sharp					No	Endoscopic
Baker 1989	USA	F	0.9	Toy	Larynx	Spherical			2.6	Pink	No	Endoscopic
Bakhshaee 2012	Iran	M	12	Whistle	Bronchus	Conical	2			Red	No	Endoscopic
Bakshi 2007	India	F	3	Spring	Larynx	Spiral				Grey	No	Endoscopic
Bakshi 2016	India	F	4	Button battery	Nose	Round				Grey	No	Endoscopic
Baliarsingh 2017	India	F	12	Safety pin	Larynx	Sharp				Grey	No	Endoscopic
Barrett 1995	UK	F	0.3	Dummy	Pharynx	Round				Multicolor	No	Endoscopic
Başok 1997	Turkey	M	8	Grass	Lungs	Elongated				Green	No	Endoscopic
Başok 1997	Turkey	M	7	Grass	Bronchus	Elongated				Green	No	Surgical
Başok 1997	Turkey	F	6	Grass	Bronchus	Elongated	3	1		Green	No	Surgical
Bhat 1996	UK	M	1	Nail	Larynx	Round	2			Pink	No	Endoscopic
Boleken 2005	Turkey	F	2	Stone	Bronchus	Round			0.3	Grey	No	Endoscopic
Bradshaw 2019	USA	M	1	Nail	Bronchus	Sharp	2.2			Grey	No	Endoscopic
Brand 2003	Netherlands	M	8	Needle	Bronchus	Sharp				Grey	No	Endoscopic

Brown 1994	New Zealand	M	5	Button battery	Nose	Round			1.2	Grey	No	Surgical
Bukhari 2022	Saudi Arabia	F	0.9	Glass piece	Larynx	Oval				Grey	No	Endoscopic
Cakir 2012	Turkey	F	12	Wire	Bronchus	Sharp	2			Black	No	Endoscopic
Capo 1986	USA	M	5	Button battery	Nose	Round				Grey	No	Surgical
Chang 2015	Taiwan	M	7	Ant	Pharynx	Elongated	1	0.6		Black	No	Endoscopic
Chaudhry 2020	Saudi Arabia	M	5	Led	Lungs	Oval				Brown	No	Endoscopic
Chen 2018	China	M	0.8	Film	Pharynx	Polygon	2	2		Trasparent	No	Endoscopic
Chhangani 1966	India	M	12	Spring	Larynx	Spiral				Grey	No	Endoscopic
Choy 1996	USA	M	3	Sand	Bronchus	Irregular				Brown	No	Endoscopic
Chua 2006	Singapore	F	4	Button battery	Nose	Round				Grey	No	Surgical
Chua 2006	Singapore	M	4	Button battery	Nose	Round				Grey	No	Surgical
Chua 2006	Singapore	M	4	Button battery	Nose	Round				Grey	No	Surgical
Cleveland 1998	UK	M	8	Wedge	Lungs	Irregular	1.5	0.7		Yellow	No	Surgical
Concerto 2018	Italy	M	0.8	Snack wrapping	Larynx	Cylindrical		0.8		Red	No	Endoscopic
Concerto 2018	Italy	M	0.8	Toothbrush cover	Larynx	Polygon				Trasparent	No	Endoscopic
Daines 2008	USA	F	12	Pen	Bronchus	Cylindrical					No	Endoscopic
Datema 2009	Netherlands	F	14	Needle	Bronchus	Sharp				Grey	No	Endoscopic
Dave 2007	India	F	12	Needle	Larynx	Sharp				Grey	No	Endoscopic
Davis 2007	UK	M	1	Tube	Trachea	Cylindrical				Black	No	Endoscopic
Davis 2019	USA	M	3	Button	Larynx	Round			1	Trasparent	No	Surgical
DeJesusBarbosa 2016	Brazil	F	7	Pen	Bronchus	Cylindrical			2	Black	No	Endoscopic
Deng 2010	China	M	7	Iron wire	Larynx	Elongated				Grey	No	Endoscopic
Deng 2010	China	M	9	Button	Larynx	Round				White	No	Endoscopic
Deng 2010	China	F	1	Metal piece	Pharynx	Cylindrical			1.8	Grey	No	Endoscopic
Deng 2016	China	M	6	Nail	Bronchus	Sharp				Grey	No	Surgical
Dinleyici 2008	Turkey	M	1	Toy	Trachea	Oval	1.4	0.9		White	No	Endoscopic
Dorfman 2011	USA	M	0.5	Toy	Pharynx	Sharp					No	Surgical
Doyle 2009	UK	M	0.7	Magazine cover piece	Larynx	Irregular				Pink	No	Endoscopic
Eghtedari 2003	Iran	M	8	Plastic tree piece	Pharynx	Oval	3	2.5		Green	No	Endoscopic
Eun 1984	South Korea	F	2	Sponge	Nose	Round	1	1		Yellow	No	Endoscopic
Fang 2016	China	F	11	Bead	Bronchus	Spherical			1	White	No	Endoscopic
Feng 2016	China	F	5	Stone	Bronchus	Round	0.4	0.4		Brown	No	Endoscopic
Findlay 2003	UK	F	3	Plastic tree piece	Bronchus	Cylindrical	1		0.6	Red	No	Endoscopic
Fosarelli 1988	USA	F	2	Button battery	Nose	Round			1	Grey	No	Surgical
Fraccaroli 2023	Italy	M	1	Tablet package	Larynx	Irregular	1.5	1		Grey	No	Endoscopic
Fraga 2002	USA	M	8	Pen	Bronchus	Cylindrical	1.2	0.5		Blue	No	Surgical
Freiman 2001	USA	M	8	Toy	Bronchus	Cylindrical	1.2		0.8	Yellow	No	Endoscopic
Garg 2010	USA	M	7	Coin	Trachea	Round				Grey	No	Surgical
Gerber 2020	USA	M	0.2	Baby wipe	Larynx	Irregular				White	No	Endoscopic
Gibson 2000	USA	M	2	Bead	Bronchus	Spherical				Red	No	Endoscopic
Gilbert 1970	UK	F	5	Safety pin	Larynx	Sharp				Grey	No	Surgical
Goez 1994	Israel	F	1	Particles	Bronchus	Spherical	4				No	Endoscopic

Goh 2015	China	M	0.7	Plastic wrapper	Larynx	Irregular	1.4	1.2		Multicolor	No	Endoscopic
Gomez-Acevedo 2010	Mexico	F	1	Ketchup envelope	Pharynx	Irregular	2.5			Red	No	Naturalis
Gómez-Ramos 2022	Mexico	M	12	Plastic spray cup	Larynx	Cylindrical	2		1	Green	No	Surgical
Goswami 2016	India	F	4	Toothbrush cover	Pharynx	Elongated	5.4	1.8		Yellow	No	Endoscopic
Goussard 2020	South Africa	M	12	Needle	Larynx	Sharp				Grey	No	Endoscopic
Goussard 2021	South Africa	M	1	Button battery	Pharynx	Round				Grey	No	Endoscopic
Goyal 2007	India	M	2	Nail	Bronchus	Sharp	3		0.7	Black	No	Endoscopic
Gupta 2010	India	M	4	Blade	Larynx	Polygon				Grey	No	Endoscopic
Hada 2012	India	M	7	Ball bearing	Bronchus	Spherical			0.8		No	Endoscopic
Hainer 2016	Germany	F	10	Needle	Bronchus	Sharp			3		No	Surgical
Haloob 2014	UK	M	1	Wall plug	Trachea	Cylindrical				Red	No	Endoscopic
Hamidi 2022	Afghanistan	M	1	Can tab	Pharynx	Round				Grey	No	Endoscopic
Hemead 2021	Egypt	M	1	Leaf	Bronchus	Elongated	3.8			Yellow	No	Endoscopic
Heyworth 2019	Australia	F	0.8	Ornamental object	Pharynx	Sharp	4			Pink	No	Endoscopic
Hickey 2022	Canada	M	1	Thumbtack	Larynx	Sharp	2.3	0.1		Black	Yes	Surgical
Hiebert 2016	USA	F	5	Coin	Trachea	Round			2	Brown	No	Endoscopic
Hilman 1980	USA	M	7	Spike	Lungs	Sharp	3	1		Black	No	Surgical
Hootnick 2015	USA	M	1	Glass piece	Larynx	Polygon					No	Endoscopic
Hosokawa 2022	Israel	F	1	Toy	Larynx	Irregular	1	1		Gold	No	Endoscopic
Humphries 1988	USA	M	1	Ornamental object	Lungs	Cylindrical	1		0.3	Orange	Yes	Surgical
Hussain 1994	UK	M	0.5	Safety pin	Larynx	Sharp				Grey	No	Endoscopic
Idris 2018	Canada	M	13	Nail	Bronchus	Sharp	1.4			Grey	No	Endoscopic
Iqbal 2011	India	F	10	Tube	Bronchus	Cylindrical				White	No	Surgical
Issaka 2023	Ghana	M	4	Needle	Lungs	Sharp				Grey	No	Surgical
Jabbardarjani 2009	Iran	M	5	Bead	Lungs	Oval				Blue	No	Endoscopic
Jain 2013	India	M	4	Pen	Trachea	Conical	1.2			Blue	No	Endoscopic
Jardeleza 2021	Australia	M	2	Wand	Pharynx	Elongated	1.4			Brown	No	Surgical
Jayaraj 2017	Canada	M	1	Nail	Bronchus	Sharp	5	0.2		Brown	No	Endoscopic
Jean 2022	Malaysia	M	6	Toy	Bronchus	Cylindrical	1.5		1	White	No	Surgical
Jotdar 2015	India	M	3	Ring	Nose	Round			1	Gold	No	Endoscopic
Jotdar 2016	Iran	M	2	Cap	Pharynx	Round	2		2	White	No	Endoscopic
Kansal 2015	India	M	0.8	Toy	Larynx	Irregular					No	Endoscopic
Kansal 2015	India	M	2	Adhesive	Trachea	Round				Trasparent	No	Endoscopic
Kansal 2015	India	M	3	Stone	Trachea	Irregular					No	Endoscopic
Kara 2022	Turkey	M	0.9	Plastic piece	Larynx	Irregular	0.8	0.1		White	No	Endoscopic
Kasuka 2022	Uganda	F	7	Leech	Pharynx	Cylindrical	3			Black	No	Endoscopic
Kathuria 2017	India	M	3	Blade	Bronchus	Polygon	1.1	0.6	0.1	Grey	No	Endoscopic
Katz 1979	USA	M	4	Cotton piece	Nose	Irregular				White	No	Surgical
Katz 1979	USA	F	2	Cotton piece	Nose	Irregular				White	No	Surgical
Katz 1979	USA	M	8	Cotton piece	Nose	Irregular				White	No	Surgical
Kawano 2007	Japan	F	1	Wand	Pharynx	Elongated	2	0.4		Yellow	No	Surgical
Kazi 2015	Pakistan	M	1	Nail	Pharynx	Sharp	5			Grey	No	Endoscopic
Kent 1990	UK	F	6	Toy	Larynx	Irregular	2				No	Surgical

Kent 1990	UK	M	1	Screw	Larynx	Cylindrical					No	Surgical
Kent 1990	UK	F	1	Screw	Larynx	Cylindrical					Yes	Surgical
Kim 2021	South Korea	F	10	Pin	Lungs	Sharp	3.3			Red	No	Endoscopic
Kondo 2006	Japan	M	0.5	Candy paper	Bronchus	Polygon	1.5	3		Trasparent	No	Endoscopic
Kumar 2003	India	F	0.7	Hook	Larynx	Irregular	1			Black	No	Endoscopic
Kumar 2013	India	F	12	Safety pin	Larynx	Elongated				Grey	No	Endoscopic
Kumar 2013	India	M	6	Spring	Larynx	Spiral				Grey	No	Surgical
Kumar 2013	India	M	2	Screw	Pharynx	Polygon	2.5	1		Brown	No	Surgical
Kurul 2002	Turkey	F	4	Ring	Pharynx	Round					No	Surgical
Lau 2015	China	F	1	Led	Bronchus	Irregular	2			Trasparent	No	Endoscopic
Lavarde 2003	France	M	10	Toy	Bronchus	Conical	0.7	0.4			No	Endoscopic
Leffler 2006	USA	M	6	Ball bearing	Larynx	Spherical				Grey	No	Endoscopic
Leiberman 1985	Israel	M	2	Cellophane	Nose	Irregular	3.5	1		Trasparent	No	Surgical
Leonard 2015	USA	M	0.1	Tube	Bronchus	Cylindrical	3.2			Trasparent	Yes	Surgical
Liao 2015	China	F	1	Button battery	Trachea	Round				Grey	No	Endoscopic
Lima 2017	Italy	F	13	Fishing line	Bronchus	Sharp				Grey	No	Surgical
Liman 2012	Turkey	F	2	Antenna	Trachea	Elongated				Grey	No	Endoscopic
Little 2000	USA	M	0.4	Cap	Larynx	Cylindrical	3			Trasparent	No	Endoscopic
Lloyd-Thomas 1986	UK	M	1	Button	Trachea	Round			1	Grey	No	Endoscopic
Loh 2003	Singapore	M	3	Button battery	Nose	Round				Grey	No	Surgical
Loh 2003	Singapore	M	4	Button battery	Nose	Round				Grey	No	Surgical
Loh 2003	Singapore	M	4	Button battery	Nose	Round				Grey	No	Surgical
Loh 2003	Singapore	M	2	Button battery	Nose	Round				Grey	No	Surgical
Loh 2003	Singapore	M	3	Button battery	Nose	Round				Grey	No	Surgical
Łoś-Rycharska 2021	Poland	M	10	Wire	Bronchus	Sharp	3			Grey	No	Endoscopic
Ludemann 2007	Canada	M	12	Thumbtack	Bronchus	Sharp	3	0.5		Trasparent	No	Endoscopic
Ludemann 2007	Canada	M	15	Dart	Larynx	Sharp	8	1		Black	No	Endoscopic
Ludemann 2007	Canada	F	11	Pin	Bronchus	Elongated	5			Yellow	No	Endoscopic
Lukse 2014	USA	M	12	Dart	Bronchus	Cylindrical	2.8			Grey	No	Endoscopic
MacNeil 2010	USA	M	4	Twig	Pharynx	Cylindrical					No	Endoscopic
MacNeil 2010	USA	F	3	Plastic piece	Pharynx	Irregular				Trasparent	No	Endoscopic
MacNeil 2010	USA	F	3	Bark	Pharynx	Irregular				Brown	No	Endoscopic
MacNeil 2010	USA	F	0.9	Twig	Pharynx	Cylindrical				Brown	No	Endoscopic
Maglione 2022	Italy	M	1	Tablet	Bronchus	Spherical			0.6		No	Endoscopic
Maguire 2012	UK	F	6	Tube	Bronchus	Cylindrical			1	Brown	No	Surgical
Majd 1977	USA	M	2.2	Screw	Bronchus	Sharp	1				No	Endoscopic
MarínGiraldo 2023	Colombia	F	1	Screw	Bronchus	Sharp				Grey	No	Surgical
Marks 1993	USA	M	0.7	Electronic circuit	Lungs	Elongated				Trasparent	No	Surgical
Marzabadi 2012	New Zealand	M	5	Pen	Bronchus	Irregular				Trasparent	No	Endoscopic
Mathur 2011	Nepal	M	1	Nail	Trachea	Sharp	4.5	2		Brown	Yes	Surgical
Mayr 1997	Austria	M	3	Screw	Bronchus	Spiral	6.5			Grey	No	Endoscopic
McAfee 2011	UK	M	15	Pen	Bronchus	Conical	1.7	0.5		Trasparent	No	Endoscopic
Mehta 2012	India	M	4	Whistle	Bronchus	Cylindrical				Red	No	Endoscopic

Mekonnen 2013	Ethiopia	M	7	Leech	Trachea	Elongated	6			Brown	No	Endoscopic
Mellema 1995	USA	M	6	Sand	Trachea	Spherical				Brown	No	Endoscopic
Mills 1977	USA	M	1	Pin	Lungs	Sharp				Grey	No	Endoscopic
Montazeri 2009	Iran	M	11	Leech	Pharynx	Elongated	2	3		Brown	No	Surgical
Morais 2021	Portugal	F	2	Blade	Trachea	Elongated				Grey	No	Endoscopic
Morgenstein 1970	USA	M	2	Chain	Trachea	Elongated	7	0.1		Grey	No	Endoscopic
Morrison 1966	UK	M	8	Pen	Lungs	Conical					No	Endoscopic
Morrison 1966	UK	M	9	Pen	Lungs	Conical				Red	No	Endoscopic
Moskowitz 1982	USA	F	1	Plat piece	Larynx	Irregular	0.8	0.9			No	Endoscopic
Moskowitz 1982	USA	F	1	Plat piece	Larynx	Irregular					No	Endoscopic
Moss 1986	USA	F	10	Ball bearing	Bronchus	Spherical			0.5	Grey	No	Endoscopic
Ms 2023	India	M	5	Pen	Trachea	Conical				Blue	No	Endoscopic
Mülazımoğlu 2014	Turkey	F	11	Pen	Nose	Conical	5			Black	No	Endoscopic
Mundra 2014	India	F	0.5	Scarp piece	Larynx	Irregular	1	0.5		Black	No	Endoscopic
Mundra 2014	India	F	0.3	Ring	Larynx	Irregular	1.5	0.8		Gold	No	Endoscopic
Mundra 2014	India	M	0.5	Stone	Bronchus	Irregular	1	0.5		Grey	No	Endoscopic
Mundra 2014	India	M	0.5	Stone	Bronchus	Irregular	2.5	1		Black	No	Endoscopic
Munjal 2000	Slovenia	M	5	Spring	Pharynx	Spiral				Grey	No	Endoscopic
Murata 1996	Japan	M	1	Nettle	Bronchus	Irregular	4			White	No	Endoscopic
Nambirajan 2001	India	M	2	Needle	Bronchus	Sharp				Grey	No	Surgical
Nanda 2020	USA	M	5	Magnets	Bronchus	Cylindrical	2			Black	No	Endoscopic
Nasr 2005	Canada	M	1	Grass	Lungs	Elongated	5.8			Green	No	Endoscopic
Nasr 2005	Canada	M	8	Grass	Lungs	Elongated	2.7	0.5		Green	No	Endoscopic
Navalakhe 1994	India	M	10	Whistle	Lungs	Elongated	2.8	0.6		Black	No	Endoscopic
Newson 1998	USA	M	0.6	Grass	Lungs	Elongated	4	1.3		Brown	No	Surgical
Nozaki 2003	Japan	M	2	Wand	Pharynx	Elongated	4			Brown	No	Surgical
Pace-Asciak 2009	Canada	M	5	Button	Bronchus	Round			0.8	Grey	No	Surgical
Palmer 2006	USA	F	9	Toy	Larynx	Irregular	1.5	0.8		Trasparent	No	Surgical
Parker 2016	UK	M	13	Button battery	Nose	Round		0.7	1.2	Grey	No	Surgical
Petrovic 2012	Serbia	M	0.9	Plastic piece	Bronchus	Oval	1	0.3			No	Endoscopic
Philip 2004	Australia	M	2	Sticker	Larynx	Irregular	2				No	Endoscopic
Poudyal 2021	Nepal	F	1	Nail	Bronchus	Sharp	2		0.5	Grey	No	Naturalis
Punnoose 2019	Emirates	F	1	Ornamental object	Larynx	Sharp	1			Blue	No	Endoscopic
Rahim 2013	Malaysia	M	9	Needle	Trachea	Sharp	2			Red	No	Endoscopic
Rashid 2015	USA	M	9	Leaves	Bronchus	Irregular	3				No	Surgical
Ravikumar 2020	India	M	1	Pen	Pharynx	Cylindrical				Trasparent	No	Surgical
Razafimanjato 2021	Madagascar	F	11	Pen	Bronchus	Cylindrical				Red	No	Surgical
Ren 2019	China	F	1	Chain	Trachea	Sharp	9	0.6		Grey	No	Surgical
Ren 2021	China	M	2	Magnets	Trachea	Round			0.5	Multicolor	No	Naturalis
Richard 2021	France	F	1	Spike (Hordenum marinum)	Larynx	Elongated					No	Surgical
Roberts 2008	USA	M	2	Safety pin	Bronchus	Sharp				Grey	No	Endoscopic
Rosenthal 2018	USA	F	6	Elastic	Larynx	Round			2	Yellow	No	Endoscopic
Ross 1988	USA	M	0.8	Spring	Bronchus	Spiral				White	No	Endoscopic
Ross 2000	UK	F	3	Fishing roll bell	Trachea	Spherical			1		No	Endoscopic

Ruangnapa 2021	Thailand	F	0.5	Dart	Trachea	Conical	1.5		2	Pink	No	Endoscopic
Ruegemer 1999	USA	M	8	Ball bearing	Lungs	Spherical			0.5	Black	No	Endoscopic
Sahni 2002	India	M	2	Nail	Bronchus	Sharp	5.5			Black	No	Endoscopic
Sakamoto 2022	Japan	F	1	Coin	Pharynx	Round				Red	No	Endoscopic
Samdhani 2023	Germany	M	4	Led	Bronchus	Elongated				Trasparent	No	Surgical
Samdhani 2023	Germany	M	0.7	Hairpin	Bronchus	Elongated				Grey	No	Surgical
Samra 2018	USA	M	10	Toy	Larynx	Irregular					No	Surgical
Samra 2018	USA	M	9	Pen	Larynx	Cylindrical	2.3		0.5		No	Surgical
Sarafi 2022	Iran	M	7	Magnets	Bronchus	Cylindrical	2.6			Black	No	Surgical
Scerbo 2019	Canada	M	13	Pin	Trachea	Sharp					No	Endoscopic
Sedhai 2022	Nepal	F	8	Safety pin	Pharynx	Sharp				Grey	No	Endoscopic
Sembiring 1995	Indonesia	M	8	Pen	Bronchus	Elongated					No	Endoscopic
Senthilraj 2019	Bangladesh	M	8	Thumbtack	Bronchus	Sharp	2.1			White	No	Endoscopic
Seth 2017	India	M	7	Button battery	Nose	Round			1.1	Grey	No	Surgical
Shad 2012	India	F	1	Safety pin	Bronchus	Sharp				Grey	No	Endoscopic
Shamon 2011	Canada	M	2	Adhesive	Larynx	Round	1	1		Green	No	Endoscopic
Sharma 1996	India	M	9	Beetle	Lungs	Irregular	3	1.5		Black	No	Endoscopic
Sharma 1999	Malaysia	F	1	Pen	Larynx	Conical	1.3			Green	No	Endoscopic
Sharma 2006	India	M	12	Whistle	Bronchus	Elongated				Grey	No	Endoscopic
Sharma 2009	India	F	6	Bead	Bronchus	Oval			1.2	Blue	No	Surgical
Sharma 2019	India	F	8	Ring	Larynx	Round				Grey	No	Surgical
Shikada 2007	Japan	F	9	Toy	Bronchus	Elongated	4	0.2	0.2	Grey	No	Endoscopic
Shivakumar 2004	India	M	12	Coal piece	Bronchus	Irregular	2	1		Black	No	Endoscopic
Shivakumar 2004	India	F	3	Pen	Bronchus	Irregular					No	Endoscopic
Shuaib 2014	USA	M	6	Toy	Bronchus	Polygon	4				No	Endoscopic
Sigalet 1988	Canada	M	0.3	Button battery	Lungs	Round				Grey	No	Surgical
Simpson 2020	USA	M	11	Thumbtack	Bronchus	Sharp	1.3			Yellow	No	Endoscopic
Singh 1999	India	M	0.1	Blade	Larynx	Elongated	2.5	1.5		Grey	No	Endoscopic
Singh 1999	India	M	0.1	Hook	Larynx	Sharp				Grey	No	Endoscopic
Skinner 1986	UK	M	12	Button battery	Nose	Round				Grey	No	Surgical
Sobin 2017	USA	M	10	Bead	Larynx	Spherical	1.3	0.7		Orange	No	Endoscopic
Somerville 2004	UK	F	2	Plastic piece	Trachea	Round	2			White	No	Surgical
Spencer 1981	USA	F	4	Grass	Bronchus	Sharp	3.5	1		Green	No	Endoscopic
Spencer 1981	USA	M	1	Grass	Bronchus	Sharp				Green	No	Endoscopic
SultanAbdulKader 2023	Malaysia	F	1	Plastic piece	Larynx	Polygon	1	1		Trasparent	No	Surgical
Sunkum 2011	Libya	F	2	Dart	Pharynx	Cylindrical				Grey	No	Naturalis
Suryanarayana 1994	India	M	5	Screw	Bronchus	Elongated	2			Grey	No	Endoscopic
Swain 2016	India	M	2	Metal piece	Pharynx	Irregular					No	Endoscopic
SzabÃ³ 2021	Hungary	M	3	Grass	Lungs	Elongated	5			Green	No	Naturalis
Szczupak 2019	USA	F	1	Toy	Larynx	Polygon				Multicolor	No	Endoscopic
Talha 2021	Bangladesh	M	4	Pellet	Lungs	Spherical				Red	No	Surgical
Tan 1991	Singapore	M	1	Safety pin	Larynx	Elongated				Grey	No	Endoscopic
Tang 2014	Malaysia	F	6	Whistle	Larynx	Cylindrical	1.8		1.2	Trasparent	No	Surgical
Tariq 2012	UK	F	14	Pin	Lungs	Elongated				Grey	No	Endoscopic

Taylor 2020	USA	M	2	Magnets	Larynx	Spherical				Grey	No	Endoscopic
Thamboo 2008	Canada	M	0.7	Ornamental object	Larynx	Irregular				Green	No	Endoscopic
Thamboo 2008	Canada	M	1	Adhesive	Bronchus	Irregular				Multicolor	No	Endoscopic
Thompson 1979	Zimbawe	F	10	Toy	Bronchus	Cylindrical					No	Endoscopic
Thornton 2015	Canada	M	2	Thumbtack	Bronchus	Sharp	2.3	0.5		Blue	No	Endoscopic
Thornton 2015	Canada	M	15	Thumbtack	Lungs	Sharp	2.3	0.5		Blue	No	Endoscopic
Thornton 2015	Canada	M	9	Thumbtack	Bronchus	Sharp	2.3	0.5		Blue	No	Endoscopic
Tong 2021	Australia	M	1	Magnets	Bronchus	Spherical			0.6	Grey	No	Endoscopic
Tsang 2017	Ireland	M	7	Toy	Bronchus	Round	0.5	0.7		White	No	Endoscopic
Tseng 1996	China	M	5	Toy	Bronchus	Cylindrical			0.6	Yellow	No	Endoscopic
Ülk 2008	Turkey	M	6	Stone	Trachea	Spherical	2.7	1.3		Grey	No	Endoscopic
Vahidi 2020	USA	M	11	Splinter	Pharynx	Sharp	5			Grey	No	Endoscopic
VanDyke 1976	USA	M	12	Sand	Bronchus	Irregular			0.2		No	Surgical
Vas 2000	India	F	11	Tube	Trachea	Cylindrical	1		0.2		No	Endoscopic
Veena 2015	India	M	5	Safety pin	Larynx	Elongated				Grey	No	Surgical
Verma 2012	India	M	1	Toy	Pharynx	Irregular	2			Yellow	No	Endoscopic
Vlahova 2023	Bulgaria	M	9	Spike (Hordenum marinum)	Lungs	Elongated				Green	No	Surgical
Walz 2013	USA	M	15	Dart	Trachea	Sharp	5	0.1		White	No	Endoscopic
WanDraman 2019	Malaysia	M	5	Button battery	Nose	Round				Grey	No	Endoscopic
WanDraman 2019	Malaysia	F	3	Button battery	Nose	Round				Grey	No	Endoscopic
WanDraman 2019	Malaysia	F	3	Button battery	Nose	Round				Grey	No	Endoscopic
Wang 2018	China	F	8	Bead	Bronchus	Oval			1	Blue	No	Endoscopic
Wankhede 2017	India	M	8	Bead	Bronchus	Oval				Blue	No	Endoscopic
Weston 1965	USA	M	0.5	Pacifier	Pharynx	Round				Yellow	Yes	Surgical
Weston 1965	USA	M	1	Screw	Pharynx	Sharp	1.8		0.4	Brown	Yes	Surgical
Weston 1965	USA	M	0.5	Cap	Larynx	Round		1.5	1.8	White	Yes	Surgical
Weston 1965	USA	M	1	Button	Trachea	Round	5		0.5	White	Yes	Surgical
Weston 1965	USA	F	1	Screw	Trachea	Sharp	0.5		0.2	Grey	Yes	Surgical
Weston 1965	USA	M	6	Ballon	Trachea	Round	7		1.5		Yes	Surgical
Weston 1965	USA	M	0.2	Funnel	Trachea	Conical	3		1	Green	Yes	Surgical
Wilkinson 1992	Australia	F	2	Toy	Bronchus	Sharp	4			Brown	No	Endoscopic
Wineski 2020	USA	M	2	Sticker	Pharynx	Irregular	3	2.5		Multicolor	No	Endoscopic
Wong 2002	Taiwan	M	5	Spring	Bronchus	Spiral	2			Grey	No	Endoscopic
Wong 2019	USA	F	0.8	Candy paper	Lungs	Irregular				Trasparent	No	Endoscopic
Wu 2019	China	F	8	Whistle	Lungs	Cylindrical	1.2		0.5	Red	No	Endoscopic
Xu 2015	China	M	9	Magnets	Bronchus	Oval	3		0.9	Grey	No	Surgical
Yang 2018	China	F	1	Chain	Trachea	Irregular	9	0.6		Yellow	No	Surgical
Yeung 2017	USA	M	1	Led	Bronchus	Cylindrical	1.2			Trasparent	No	Endoscopic
Yogev 2021	Israel	M	2	Magnets	Larynx	Spherical			0.5	Grey	No	Endoscopic
Zeitlin 2000	USA	F	6	Bead	Larynx	Spherical			0.7	Grey	No	Endoscopic
Zhao 2015	China	M	7	Leaf	Bronchus	Oval	2			Green	No	Endoscopic

Table S4. List of references of the articles included in the review

1. Abbas, Q.; Saeed, A.A.; Sarwar, M.; Khan, I.; Ahmed, W.; Sultana, N. Magnet-Related Foreign Body Aspiration in Two Children Requiring Thoracotomy: A Concerning Report. Journal of Pediatric Surgery Case Reports 2022, 79, doi:10.1016/j.epsc.2022.102230.
2. Abder-Rahman, H.A. Infants Choking Following Blind Finger Sweep. J Pediatr (Rio J) 2009, 85, 273–275, doi:10.2223/JPED.1892.
3. Abraham, Z.S.; Kahinga, A.A.; Mapondella, K.B.; Massawe, E.R.; Ntunaguzi, D. Spontaneous Expulsion of an Intrabronchial Sharp Metallic Foreign Body and Migration to the Gastrointestinal Tract at Muhimbili National Hospital: Case Report and Literature Review. International Journal of Surgery Case Reports 2020, 72, 423–425, doi:10.1016/j.ijscr.2020.05.100.
4. Adoga, A.A.; Kokong, D.D.; Ma'an, N.D. Endobronchial Metallic Foreign Body in a Nigerian Child: Management Difficulties and the Need for Caution: A Case Report. Cases J 2009, 2, 7766, doi:10.4076/1757-1626-2-7766.
5. Ahad, A.; Majid, A.; Ahmad, N.; Manhas, N. An Unusual Tracheal Foreign Body - a Case Report. Indian J Otolaryngol Head Neck Surg 1999, 52, 100–101, doi:10.1007/BF02996453.
6. Aihole, J.S. Spontaneous Expulsion of an Unusual Sharp Metallic Foreign Body: A Rare Occurrence. Indian Journal of Otolaryngology and Head and Neck Surgery 2019, 71, 820–822, doi:10.1007/s12070-019-01648-4.
7. Aihole, J.S. Stridor in a Child: It's Diagnostic Challenges. Respir Med Case Rep 2020, 29, 101011, doi:10.1016/j.rmcr.2020.101011.
8. Akhter, J.; Gaspar, M.; Bassuk, A.; Roberts, J. Inadvertent Removal of Foreign Body via a Flexible Fiberoptic Bronchoscope in a 4-Year-Old Boy. Pediatr Pulmonol 1994, 18, 51–52, doi:10.1002/ppul.1950180113.
9. Al-Halfawy, A. Flexible Bronchoscopy for the Retrieval of Aspirated Metallic Pins: A Case Series. Journal of Bronchology 2007, 14, 83–85, doi:10.1097/LBR.0b013e31803b946a.
10. Aljahdali, A.; Abdalwahab, A.; Al-Majed, S. Acquired Tracheoesophageal Fistula and Detachable Soda Can Tab. Journal of Pediatric Surgery Case Reports 2021, 64, doi:10.1016/j.epsc.2020.101689.
11. AlKhalifah, A.S.; AlJassim, N.A. Venovenous Extra Corporeal Life Support in an Infant with Foreign Body Aspiration: A Case Report. Respir. Med. Case Rep. 2022, 37, doi:10.1016/j.rmcr.2022.101636.
12. Alotaibi, S.M.; Alobaida, N.W.; Aljomah, D.S.; AlShahrani, M.; Binnasser, A. Removal of Large Foreign Body from Airway via Combined Endoscopic and Open Approach: A Case Report and Literature Review. Otolaryngology Case Reports 2022, 23, doi:10.1016/j.xocr.2022.100417.
13. Ambu, V.K.; Narayanan, P.; Ratnasingam, V. Neglected Laryngeal Foreign Body. J Laryngol Otol 2001, 115, 740–742, doi:10.1258/0022215011908801.
14. Anajar, S.; Ansari, R.; Hassnaoui, J.; Abada, R.; Roubal, M.; Mahtar, M. An Unusual Cause of Severe Dyspnea: A Laryngeal Live Leech: Case Report. International Journal of Surgery Case Reports 2017, 32, 9–11, doi:10.1016/j.ijscr.2016.12.007.
15. Anand, C.S.; Maru, Y.K. Neglected Iron Nail in the Larynx. Indian Journal of Otolaryngology 1979, 31, 58, doi:10.1007/BF02992230.

16. Antón-Pacheco, J.L.; Berchi, F.J. Acquired Tracheo-Esophageal Fistula in a Child Caused by an Unsuspected Esophageal Foreign Body. International Journal of Pediatric Otorhinolaryngology Extra 2008, 3, 161–164, doi:10.1016/j.pedex.2008.02.004.
17. Arun Babu, T.; Ananthakrishnan, S. Unusual Presentation of Sand Aspiration in a 14-Mo-Old Child. Indian J Pediatr 2013, 80, 786–788, doi:10.1007/s12098-012-0913-7.
18. Arutyunyan, T.; Odetola, F.O. Foreign Body in the Airway: When Imaging Is Not Enough. Clinical Pediatrics 2014, 53, 186–188, doi:10.1177/0009922813475705.
19. Asaf, B.B.; Vijay, Cl.; Bishnoi, S.; Dua, N.; Kumar, A. Thoracoscopic Foreign Body Removal and Repair of Bronchus Intermedius Following Injury during Failed Bronchoscopic Retrieval. Lung India 2017, 34, 182–184, doi:10.4103/0970-2113.201296.
20. Atmaca, S.; Gümüşsoy, M.; Tulga, T.; Bayraktar, C.; Şensoy, G. The Value of Flexible Laryngoscopy in Diagnosis of an Unusual Laryngeal Foreign Body: Spiny Cocklebur (Xanthium Spinosum). Turkiye Klinikleri Journal of Medical Sciences 2011, 31, 265–267, doi:10.5336/medsci.2009-13095.
21. Atmaca, S.; Unal, R.; Seşen, T.; Kiliçarslan, H.; Unal, A. Laryngeal Foreign Body Mistreated as Recurrent Laryngitis and Croup for One Year. Turk J Pediatr 2009, 51, 65–66.
22. Azurara, L.; Lemos, C. Migratory Pushpin in the Tracheobronchial Tree. BMJ Case Reports 2016, 2016, doi:10.1136/bcr-2016-214857.
23. Baker, M.D. Near-Miss Asphyxiation from a Toy Ball: A Small Parts Failure. Pediatr Emerg Care 1989, 5, 34–36, doi:10.1097/00006565-198903000-00011.
24. Bakhshaee, M.; Bameshki, A.R.; Irani, S. An Unusual Presentation of a Bronchial Foreign Body. Otolaryngology - Head and Neck Surgery 2012, 146, 169–170, doi:10.1177/0194599811412031.
25. Bakshi, J.; Mann, S.B.S.; Gupta, A.K. Unusual Presentation of Laryngeal Foreign Bodies - Report of Two Rare Cases. Indian Journal of Otolaryngology and Head and Neck Surgery 2007, 59, 252–254, doi:10.1007/s12070-007-0072-8.
26. Bakshi, S.S.; Coumare, V.N.; Priya, M.; Kumar, S. Long-Term Complications of Button Batteries in the Nose. Journal of Emergency Medicine 2016, 50, 485–487, doi:10.1016/j.jemermed.2015.10.041.
27. Baliarsingh, D.; Rath, A.; Hota, A.; Panigrahi, R. Open Safety Pin in Larynx: A Case Report and Review of Literature. Otorhinolaryngology Clinics 2017, 9, 21–24, doi:10.5005/jp-journals-10003-1255.
28. Barrett, T.G.; Debelle, G.D. Near-Fatal Aspiration of a Child's Dummy: Design Fault or Deliberate Injury? J Accid Emerg Med 1995, 12, 154–155, doi:10.1136/emj.12.2.154.
29. Başok, O.; Yaldiz, S.; Kilinçer, L. Bronchiectasis Resulting from Aspirated Grass Inflorescences. Scand Cardiovasc J 1997, 31, 157–159, doi:10.3109/14017439709058086.
30. Bhat, N.A.; Oates, J. An Unusual Foreign Body in the Larynx: A Case Report. J Laryngol Otol 1996, 110, 1164–1165, doi:10.1017/s0022215100136035.
31. Boleken, M.E.; Kaya, M.; Kanmaz, T.; Yücesan, S. The Tragedy of Pica: Stone Aspiration! Gazi Medical Journal 2005, 16, 144–145.
32. Bradshaw, J.; Dayan, J.E.; Collins, S.V.; Josephson, G. Playful Child, Dangerous Intruder: A Case of Silent Foreign Body Aspiration in a 13-Month-Old Boy. Clin Pediatr (Phila) 2019, 58, 1031–1033, doi:10.1177/0009922819851265.
33. Brand, P.L.P.; Rosingh, H.J. The "Wandering Needle". Pediatr Pulmonol 2003, 35, 152–154, doi:10.1002/ppul.10214.

34. Brown, C.R. Intranasal Button Battery Causing Septal Perforation: A Case Report. J Laryngol Otol 1994, 108, 589–590, doi:10.1017/s0022215100127513.
35. Bukhari DH; Kabli AF; Alharthi TS; Sendi E; Rashed AA A Rare Case of a Vocal Cord Foreign Body in an Infant: A Case Report. Cureus 2022, 14, e29213, doi:10.7759/cureus.29213.
36. Cakir, E.; Torun, E.; Uyan, Z.S.; Akca, O.; Soysal, O. An Unusual Case of Foreign Body Aspiration Mimicking Cavitary Tuberculosis in Adolescent Patient: Thread Aspiration. Ital J Pediatr 2012, 38, 17, doi:10.1186/1824-7288-38-17.
37. Capo, J.M.; Lucente, F.E. Alkaline Battery Foreign Bodies of the Ear and Nose. Archives of Otolaryngology--Head and Neck Surgery 1986, 112, 562–563, doi:10.1001/archotol.1986.03780050086016.
38. Chang, H.-M.; Chang, G.-H. An Unexpected Foreign Body: A Hypopharyngeal Ant. Otolaryngology - Head and Neck Surgery (United States) 2015, 153, 683–684, doi:10.1177/0194599815577088.
39. Chaudhry, I.U.H.; Eid, H.A.; Cheema, A.; Chaudhry, A.; Albadar, S.; Ali, M.G. Innovative Surgical Technique for Removal of Light Emitting Diode from Segmental Bronchus in a Child: After the Failure of Endoscopic Retrieval. Int J Surg Case Rep 2020, 73, 365–368, doi:10.1016/j.ijscr.2020.07.059.
40. Chen, G.; Luo, Y.; Pan, H.; Teng, Y.; Liang, Z.; Li, L. Uncommon Foreign Body in the Hypopharynx: A Case Report. Medicine (Baltimore) 2018, 97, e11242, doi:10.1097/MD.0000000000011242.
41. Chhangani, D.L.; Agarwal, K.K. An Unusual Foreign Body in the Larynx. Case Report. J Laryngol Otol 1966, 80, 974–976, doi:10.1017/s0022215100066275.
42. Choy, I.O.; Idowu, O. Sand Aspiration: A Case Report. J Pediatr Surg 1996, 31, 1448–1450, doi:10.1016/s0022-3468(96)90854-4.
43. Chua, D.Y.K.; Tan, H.K.K. Repair of Nasal Septal Perforations Using Auricular Conchal Cartilage Graft in Children: Report on Three Cases and Literature Review. Int J Pediatr Otorhinolaryngol 2006, 70, 1219–1224, doi:10.1016/j.ijporl.2005.12.021.
44. Cleveland, R.H.; Mark, E.J. Case Records of the Massachusetts General Hospital. New England Journal of Medicine 1998, 339, 1144–1151, doi:10.1056/NEJM199810153391608.
45. Concerto, A.; Cavallaro, M.; Visalli, C.; Bagnato, A.M.; Barbaro, U.; Salamone, I. Thin Laryngeal Foreign Bodies in Infants: Diagnostic Potential of MDCT. Respirol Case Rep 2018, 6, e00301, doi:10.1002/rcr2.301.
46. Daines, C.L.; Wood, R.E.; Boesch, R.P. Foreign Body Aspiration: An Important Etiology of Respiratory Symptoms in Children. J Allergy Clin Immunol 2008, 121, 1297–1298, doi:10.1016/j.jaci.2008.03.014.
47. Datema, F.R.; Borgstein, J. A New Method to Solve an Old Problem: Extraction of a Sharp Foreign Body from the Lateral Basal Part of the Bronchial Tree of a Child. International Journal of Pediatric Otorhinolaryngology Extra 2009, 4, 62–65, doi:10.1016/j.pedex.2008.06.006.
48. Dave, N.; Oak, S.N. Metallic Foreign Body in the Lung: A Case Report. J Pediatr Surg 2007, 42, 1282–1283, doi:10.1016/j.jpedsurg.2007.02.022.
49. Davis, R.J.; Stewart, C.M. Complete Glottic Obstruction by an Unusual Foreign Body. Otolaryngol Head Neck Surg 2019, 160, 935–936, doi:10.1177/0194599818824298.

50. Davis, S.J.; Madden, G.; Carapiet, D.; Nixon, M.; Dennis, S.; Pringle, M. Delayed Presentation of Paediatric Tracheal Foreign Body. Eur Arch Otorhinolaryngol 2007, 264, 833–835, doi:10.1007/s00405-007-0250-z.
51. De Jesus Barbosa, A.G.; Penha, D.; Zanetti, G.; Marchiori, E. Foreign Body in the Bronchus of a Child: The Importance of Making the Correct Diagnosis. Radiologia Brasileira 2016, 49, 340–342, doi:10.1590/0100-3984.2015.0169.
52. Deng, X.; Wang, J.; Chen, R.; Huang, P.; Liu, P.; Luo, X. A Straight Pin Foreign Body in a Child: Ingested or Aspirated? Springerplus 2016, 5, 1694, doi:10.1186/s40064-016-3335-6.
53. Deng, Z.; Qiu, J.; Bai, D.; Shi, L.; Zhao, Y.; Gong, G.; Song, Y.; Chen, Y.; Zong, X. Removal of Longstanding Laryngopharyngeal Foreign Bodies from Three Children. International Journal of Pediatric Otorhinolaryngology Extra 2010, 5, 85–88, doi:10.1016/j.pedex.2009.03.004.
54. Dinleyici, E.C.; Kocak, K.; Ilhan, H.; Ozkan, R.; Tutuncu, R.; Bor, O. Subcutaneous Emphysema, Pneumomediastinum and Epidural Emphysema in a Child Due to Foreign Body Aspiration Mimicking Croup Syndrome. International Journal of Pediatric Otorhinolaryngology Extra 2008, 3, 35–38, doi:10.1016/j.pedex.2007.09.005.
55. Dorfman, A.; Pauze, D.; Tilney, P. A near Fatality in a 6-Month-Old Boy from an Aspirated Toy. Air Med J 2011, 30, 64–67, doi:10.1016/j.amj.2011.01.002.
56. Doyle, C.; McDonald, D. Life! Death! Prizes! Tracheal Obstruction! Anaesthesia 2009, 64, 1390, doi:10.1111/j.1365-2044.2009.06072.x.
57. Eghtedari, F. Long Lasting Nasopharyngeal Foreign Body. Otolaryngology - Head and Neck Surgery 2003, 129, 293–294, doi:10.1016/S0194-5998(03)00471-6.
58. Eun, H.C.; Kim, K.H.; Lee, Y.S. Unusual Body Odour Due to a Nasal Foreign Body in a Child. Journal of Dermatology 1984, 11, 501–503, doi:10.1111/j.1346-8138.1984.tb01515.x.
59. Fang, N.; Sun, L.; Zhang, Y.; Zhu, W.; Wang, X. Combined Application of Laryngoscopy and Flexible Bronchoscopy in Endobronchial Foreign Body Extraction. Minim Invasive Ther Allied Technol 2016, 25, 351–354, doi:10.1080/13645706.2016.1204320.
60. Feng, S.; Liu, D.; Huang, Z.; Zhong, J. An Unusual Respiratory Tract Foreign Body: A Case Report of Pediatric Broncholithiasis. International Journal of Pediatric Otorhinolaryngology Extra 2016, 13, 50–52, doi:10.1016/j.pedex.2016.05.001.
61. Findlay, C.A.; Morrissey, S.; Paton, J.Y. Subcutaneous Emphysema Secondary to Foreign-Body Aspiration. Pediatr Pulmonol 2003, 36, 81–82, doi:10.1002/ppul.10295.
62. Fosarelli, P.; Feigelman, S.; Pearson, E.; Calimano-Diaz, A. An Unusual Intranasal Foreign Body. Pediatr Emerg Care 1988, 4, 117–118, doi:10.1097/00006565-198806000-00007.
63. Fraccaroli F; Tosini D; Lerario G; Battilocchi L; D'Onghia A; Cantarella G; Pignataro L; Neri S; Gaffuri M A Misdiagnosed Laryngeal Foreign Body Causing Recurrent Wheezing in a Pediatric Patient: Case Report. Clin Case Rep 2023, 11, e7476, doi:10.1002/ccr3.7476.
64. Fraga, J.C.; Neto, A.M.; Seitz, E.; Schopf, L. Bronchoscopy and Tracheotomy Removal of Bronchial Foreign Body. J Pediatr Surg 2002, 37, 1239–1240, doi:10.1053/jpsu.2002.34493.
65. Freiman, M.A.; McMurray, J.S. Unique Presentation of a Bronchial Foreign Body in an Asymptomatic Child. Ann Otol Rhinol Laryngol 2001, 110, 495–497, doi:10.1177/000348940111000601.
66. Garg, R.; Pandey, R.; Khanna, P.; Narang, D. Airway Management of Undiagnosed Tracheoesophageal Fistula Detected Accidentally Intraoperatively. Paediatr Anaesth 2010, 20, 970–972, doi:10.1111/j.1460-9592.2010.03413.x.

67. Gerber, N.; Iyer, S.; Parra, D.M.; Legano, L.; Tunik, M. Intentional Asphyxiation Using Baby Wipes: A Case Report and Review of the Literature. Pediatr Emerg Care 2020, 36, e397–e398, doi:10.1097/PEC.0000000000002159.
68. Gibson Jr., W.S.; Vrabec, D.P. Encounters with Challenging Bronchial Foreign Bodies: Impromptu Adaptation of Technique. Annals of Otology, Rhinology and Laryngology 2000, 109, 86–88, doi:10.1177/000348940010900116.
69. Gilbert, T.J. An Unusual Case of Inhaled Foreign Body. Anaesthesia 1970, 25, 391–392, doi:10.1111/j.1365-2044.1970.tb00227.x.
70. Goez, H.; Siplovich, L. Pneumomediastinum with Subcutaneous Emphysema Following Foreign Body Aspiration. Pediatric Surgery International 1994, 9, 513, doi:10.1007/BF00179455.
71. Goh, B.S.; Maryam, M.F.; Jaafar, M.J.; Rusydi, W. Supraglottic Foreign Body Aspiration: Delayed Diagnosis and Lesson Learnt. Hong Kong Journal of Emergency Medicine 2015, 22, 57–59, doi:10.1177/102490791502200109.
72. Gomez-Acevedo, H.H. Maneuver for the Recovery of a Foreign Body Causing a Complete Airway Obstruction: Illustrative Case. Pediatr Emerg Care 2010, 26, 39–40, doi:10.1097/PEC.0b013e3181c399cc.
73. Gómez-Ramos, J.J.; Marín-Medina, A.; Castillo-Cobian, A.A.; Felipe-Diego, O.G. Successful Management Foreign Body Aspiration Associated with Severe Respiratory Distress and Subcutaneous Emphysema: Case Report and Literature Review. Medicina (Kaunas) 2022, 58, doi:10.3390/medicina58030396.
74. Goswami, S. The Head of a Broken Toothbrush in the Parapharyngeal Space: A Rare Case Report. J Indian Soc Periodontol 2016, 20, 79–81, doi:10.4103/0972-124X.164763.
75. Goussard, P.; Mfingwana, L.; Morrison, J. Removal of Distal Airway Foreign Body with the Help of Fluoroscopy in a Child. Pediatr Pulmonol 2020, 55, E5–E7, doi:10.1002/ppul.24653.
76. Goussard, P.; Morrison, J.; Nel, E.; Mfingwana, L.; Andronikou, S.; Blokland, R.A.; Mukhtar, A.; Loock, J.W.; de Bruyn, G.H.M. Bilateral Vocal Fold Palsy Due to Ingested Battery in the Postcricoid Area/Proximal Esophagus. Pediatr Pulmonol 2021, 56, 2366–2369, doi:10.1002/ppul.25366.
77. Goyal, A.; Mohan, N.V.K.; Mundra, R.K. Spontaneous Expulsion of a Bronchial Foreign Body. Otolaryngology - Head and Neck Surgery 2007, 136, 324–325, doi:10.1016/j.otohns.2006.06.1274.
78. Gupta, K.; Gupta, P.K. Laryngoscopic Removal of Unusual Metallic Foreign Body of the Subglottic Region of the Larynx. Anesth Essays Res 2010, 4, 106–108, doi:10.4103/0259-1162.73517.
79. Hada, M.S.; Chadha, V.; Mishra, P.; Grover, M. "Slam Dunk": A Case Report of an Unusual Metallic Foreign Body. J Bronchology Interv Pulmonol 2012, 19, 156–158, doi:10.1097/LBR.0b013e31824edf25.
80. Hainer, F.; Klauß, W.; Wünsch, L.; Tüshaus, L. The Turban Pin Aspiration Syndrome: Awareness for a Subtype of Foreign Body Aspiration. Journal of Pediatric Surgery Case Reports 2016, 7, 28–30, doi:10.1016/j.epsc.2016.02.006.
81. Haloob, N.; Loizou, P.; Lyons, M. Inhalation of a Plastic Wall Plug: Implications of Foreign Body Characteristics in Paediatric Patients. BMJ Case Rep 2014, 2014, bcr-2013, doi:10.1136/bcr-2013-201725.

82. Hamidi H; Rastin MS A Rare Nasopharyngeal Foreign Body (Detached Beverage Can Stay-Tab): A Case Report. Radiol Case Rep 2022, 17, 4690–4693, doi:10.1016/j.radcr.2022.08.108.
83. Hemead, H.M.; El Achy, S.; Abdelrahman, M.; Abdelaziz, A. A Migrating Intrathoracic Leaf Complicated by Empyema in a Child. Journal of Pediatric Surgery Case Reports 2021, 74, doi:10.1016/j.epsc.2021.102031.
84. Heyworth, P.; Shulman, R. A Christmas Message: Be Careful of the Confetti Stars. Medical Journal of Australia 2019, 211, 510, doi:10.5694/mja2.50424.
85. Hickey, T.B.M.; Mathews, R.Z.; Pickup, M.J. Pediatric Push Pin Aspiration: Clinical, Radiologic, and Pathologic Correlation — Case Report. Egypt. J. Forensic Sci. 2022, 12, doi:10.1186/s41935-022-00295-1.
86. Hiebert, J.C.; Baranano, C.F. A Not so Lucky Penny. Otolaryngology - Head and Neck Surgery (United States) 2016, 154, 566–567, doi:10.1177/0194599815625938.
87. Hilman, B.C.; Kurzweg, F.T.; McCook, W.W.J.; Liles, A.E. Foreign Body Aspiration of Grass Inflorescences as a Cause of Hemoptysis. Chest 1980, 78, 306–309, doi:10.1378/chest.78.2.306.
88. Hootnick, J.L.; Schroeder, J.W. Glass in the Glottis: A Pediatric Case Report. International Journal of Pediatric Otorhinolaryngology Extra 2015, 10, 4–7, doi:10.1016/j.pedex.2014.10.005.
89. Hosokawa, S. An Unhappy Shooting Star: A Laryngeal Foreign Body Masquerading as Croup. Ear Nose Throat J. 2022, 101, 372–373, doi:10.1177/0145561320962585.
90. Humphries, C.T.; Wagener, J.S.; Morgan, W.J. Fatal Prolonged Foreign Body Aspiration Following an Asymptomatic Interval. Am J Emerg Med 1988, 6, 611–613, doi:10.1016/0735-6757(88)90104-0.
91. Hussain, S.S.; Raine, C.H.; Caldicott, L.D.; Wade, M.J. An Open Safety Pin in the Larynx: A Case Report. J Laryngol Otol 1994, 108, 254–255, doi:10.1017/s0022215100126453.
92. Idris, S.; Murphy, R.A.; Witmans, M.; El-Hakim, H. A Simple Management Option for Chronically Impacted Sharp Tracheobronchial Foreign Bodies in Children. J Otolaryngol Head Neck Surg 2018, 47, 26, doi:10.1186/s40463-018-0272-0.
93. Iqbal, I.; Lateef, M.; Wani, A.A.; Rafiq, S. A Rare Case of Foreign Body Bronchus: A Case Report. Indian J Otolaryngol Head Neck Surg 2011, 63, 81–82, doi:10.1007/s12070-011-0206-x.
94. Issaka A; Seidu AS; Adjeso T Late Migration of an Aspirated Foreign Body from the Lung to the Bowel: A Plausible Explanation or a Medical Mystery. A Case Report. Int J Surg Case Rep 2023, 106, 108214, doi:10.1016/j.ijscr.2023.108214.
95. Jabbardarjani, H.R.; Kiani, A.; Arab, A. Removal of Impacted Foreign Body by Bronchoscopic Modalities. Tanaffos 2009, 8, 60–64.
96. Jain, S.; Kashikar, S.; Deshmukh, P.; Gosavi, S.; Kaushal, A. Impacted Laryngeal Foreign Body in a Child: A Diagnostic and Therapeutic Challenge. Ann Med Health Sci Res 2013, 3, 464–466, doi:10.4103/2141-9248.117937.
97. Jardeleza, C.; North, H.; Taylor, P.; Lord, D.; Cheng, A. A Chopstick Snapped in Two: A Serious Pediatric Transoral Impalement Injury Requiring External Approach (Open Neck) Operative Intervention. Otolaryngology Case Reports 2021, 21, doi:10.1016/j.xocr.2021.100350.

98. Jayaraj, A.K.; Jayaraj, P.K.; Murugesh, M.; Aruchamy, S.; Yousefzadeh, A.; Siddiqui, N.T. Tracheal Foreign Body Removal Using Flexible Bronchoscope in a Pediatric Patient. American Journal of Respiratory and Critical Care Medicine 2017, 196, 1071–1072, doi:10.1164/rccm.201703-0518IM.
99. Jean, Y.H.; Mat Lazim, N. Foreign Body Aspiration: An Alarming Whistle. Vis. J. Emerg. Med. 2022, 29, doi:10.1016/j.visj.2022.101577.
100. Jotdar, A.; Dutta, M.; Kundu, S. Nasopharynx- The Secret Vault for Lost Foreign Bodies of the Upper Aerodigestive Tract. Iran J Otorhinolaryngol 2016, 28, 431–433.
101. Jotdar, A.; Dutta, M.; Mukhopadhyay, S. Nasal Foreign Body, Dislodged and Lost - Can the Adenoids Help? J Clin Diagn Res 2015, 9, MD06-07, doi:10.7860/JCDR/2015/13772.6073.
102. Kansal, B.; Swamy, K.M.; Ramesh, H.; Kumar, B. Unusual Foreign Bodies in the Respiratory Tract of Children. Indian Pediatr 2015, 52, 611–612, doi:10.1007/s13312-015-0685-z.
103. Kara, İ.; Yıldız, M.G.; Çınar, Ö.F.; Yavuz, C.; Orhan, İ.; Sağıroğlu, S. Laryngeal Foreign Body: A Rare Case Report. B-ENT 2022, 18, 77–80, doi:10.5152/B-ENT.2022.21308.
104. Kasuka, A.; Bisso, F.; Kabagenyi, F. Bloody Wiggler, a Case Report of a Leech in the Pharynx. Int. Med. Case Rep. J. 2022, 15, 507–510, doi:10.2147/IMCRJ.S379722.
105. Kathuria, B.; Arora, V.; Wadhera, R.; Singh, S. Sharpener Blade: An Unusual Tracheobronchial Sharp Foreign Body in a Child. Lung India 2017, 34, 102–103, doi:10.4103/0970-2113.197098.
106. Katz, H.P.; Katz, J.R.; Bernstein, M.; Marcin, J. Unusual Presentation of Nasal Foreign Bodies in Children. JAMA: The Journal of the American Medical Association 1979, 241, 1496, doi:10.1001/jama.1979.03290400056026.
107. Kawano, T.; Ishitoya, J.; Endou, R.; Tsukuda, M. A Infant Case of a Chopstick as a Foreign Body in the Parapharyngeal Space. Practica Oto-Rhino-Laryngologica 2007, 100, 899–903, doi:10.5631/jibirin.100.899.
108. Kazi, S.G.I.; Habib, M.I.; Afzal, B.; Khan, I.Q.; Siddiqui, E.; Aftab, M. Nail as a Foreign Body in a Neonate, an Unusual Presentation at an Unusual Age. Journal of the Pakistan Medical Association 2015, 65, 315–316.
109. Kent, S.E.; Watson, M.G. Laryngeal Foreign Bodies. The Journal of Laryngology & Otology 1990, 104, 131–133, doi:10.1017/S0022215100112058.
110. Kim, Y.C.; Kwon, S.K. Endoscopic Management of Pin Stuck into the Segmental Bronchus. Clinical Case Reports 2021, 9, doi:10.1002/ccr3.5127.
111. Kondo, E.; Tamura, K.; Akizuki, H.; Miyazaki, K.; Takeda, N.; Nakagawa, H. A Case of Tracheobronchial Foreign Body Diagnosed by Multi-Detector Row CT. Practica Oto-Rhino-Laryngologica 2006, 99, 875–880, doi:10.5631/jibirin.99.875.
112. Kumar, A.; Dubey, K.P.; Gupta, A.; Baruah, B. Removal of Open Safety Pin in Larynx: A Challenging Event for an Otolaryngologist. Online Journal of Health and Allied Sciences 2013, 12.
113. Kumar, D.; Kumar, S.; Sahni, J.K. True Glottic Foreign Body in an Infant. Indian Journal of Otolaryngology and Head and Neck Surgery 2003, 55, 32–33.
114. Kumar, S.; Singh, D.B.; Singh, A.B. A Foreign Body of the Larynx Misdiagnosed as Vocal Cord Paralysis. BMJ Case Rep 2013, 2013, doi:10.1136/bcr-2013-200154.

115. Kumar, S.; Singh, D.B.; Singh, A.B. An Unusual Nasopharyngeal Foreign Body with Unusual Presentation as Nasal Regurgitation and Change in Voice. BMJ Case Rep 2013, 2013, doi:10.1136/bcr-2013-010005.
116. Kurul, S.; Kandogan, T. Pharyngeal Foreign Body in a Child Persisting for Three Years. Emerg Med J 2002, 19, 361–362, doi:10.1136/emj.19.4.361.
117. Lau, C.T.; Lan, L.; Wong, K.; Tam, P.K.H. A Light Bulb Moment: An Unusual Cause of Foreign Body Aspiration in Children. BMJ Case Rep 2015, 2015, doi:10.1136/bcr-2015-211452.
118. Lavarde, D.; Thi, T.N.P.; Le Bourgeois, M.; De Blic, J.; Scheinmann, P. Asthma Refractory to Treatment in a 10-Years Old Boy. Revue Francaise d'Allergologie et d'Immunologie Clinique 2003, 43, 335–337, doi:10.1016/S0335-7457(03)00191-6.
119. Leffler, S. An Unusual Method for the Removal of a Foreign Body from a Child's Airway. Pediatr Emerg Care 2006, 22, 173–174, doi:10.1097/01.pec.0000202456.64978.74.
120. Leiberman, A.; Yagupsky, P.; Lavie, P. Obstructive Sleep Apnoea Probably Related to a Foreign Body. Eur J Pediatr 1985, 144, 205–206, doi:10.1007/BF00451916.
121. Leonard, J.; Jankowska, A.; Baik, M.; Kazachkov, M. Anesthetic Management of an Extremely Premature, Extremely Low-Birth-Weight Infant Undergoing Bronchoscopy for Removal of an Aspirated Foreign Body. A & A case reports 2015, 5, 185–187, doi:10.1213/XAA.0000000000000232.
122. Liao, W.; Wen, G.; Zhang, X. Button Battery Intake as Foreign Body in Chinese Children: Review of Case Reports and the Literature. Pediatric Emergency Care 2015, 31, 412–415, doi:10.1097/PEC.0000000000000134.
123. Lima, M.; Ugolini, S.; Di Salvo, N.; Libri, M.; Gargano, T.; Ruggeri, G. A Unique Case of Foreign Body Aspiration and Recurrent Pneumothorax. Pediatr Med Chir 2017, 39, 171, doi:10.4081/pmc.2017.171.
124. Liman, S.T.; Elicora, A.; Akgul, A.G.; Ozbudak, E.; Topcu, S. Radio Antenna Aspiration. Ann Thorac Surg 2012, 94, e129, doi:10.1016/j.athoracsur.2012.06.053.
125. Little, J.P.; Tunkel, D.E.; Marsh, B.R. Foreign Body Aspiration: An Unusual Complication of Antibiotic Therapy. Arch Pediatr Adolesc Med 2000, 154, 313–314, doi:10.1001/archpedi.154.3.313.
126. Lloyd-Thomas, A.R.; Bush, G.H. All That Wheezes Is Not Asthma. Anaesthesia 1986, 41, 181–185, doi:10.1111/j.1365-2044.1986.tb13177.x.
127. Loh, W.S.; Leong, J.-L.; Tan, H.K.K. Hazardous Foreign Bodies: Complications and Management of Button Batteries in Nose. Ann Otol Rhinol Laryngol 2003, 112, 379–383, doi:10.1177/000348940311200415.
128. Łoś-Rycharska, E.; Wasielewska, Z.; Nadolska, K.; Krogulska, A. A Foreign Body in the Mediastinum as a Cause of Chronic Cough in a 10-Year-Old Child with Asthma. J Asthma 2021, 58, 276–280, doi:10.1080/02770903.2019.1684515.
129. Ludemann, J.P.; Riding, K.H. Choking on Pins, Needles and a Blowdart: Aspiration of Sharp, Metallic Foreign Bodies Secondary to Careless Behavior in Seven Adolescents. Int J Pediatr Otorhinolaryngol 2007, 71, 307–310, doi:10.1016/j.ijporl.2006.10.017.
130. Lukse, R.; Winter, R.C.; Pollock, A.N. Metallic Foreign Body Aspiration: Case of a Blowgun Dart in the Left Main Stem Bronchus. Pediatr Emerg Care 2014, 30, 586–587, doi:10.1097/PEC.0000000000000200.

131. MacNeil, S.D.; Moxham, J.P.; Kozak, F.K. Paediatric Aerodigestive Foreign Bodies: Remember the Nasopharynx. J Laryngol Otol 2010, 124, 1132–1135, doi:10.1017/S0022215110000708.
132. Maglione, M.; Antonelli, F.; Orlando, C. Iron Tablet Inhalation: Not a Foreign Body Like the Others. Pediatr. Emerg. Care 2022, 38, E1696–E1697, doi:10.1097/PEC.0000000000002780.
133. Maguire, A.; Gopalakaje, S.; Eastham, K. All That Wheezes Is Not Asthma: A 6-Year-Old with Foreign Body Aspiration and No Suggestive History. BMJ Case Rep 2012, 2012, doi:10.1136/bcr-2012-006640.
134. Majd, N.S.; Mofenson, H.C.; Greensher, J. Lower Airway Foreign Body Aspiration in Children. An Analysis of 13 Cases. Clin Pediatr (Phila) 1977, 16, 13–16, doi:10.1177/000992287701600101.
135. Marín Giraldo, A.F.; Bustillos Serna, C.F.; Figueroa Gutiérrez, L.M.; Osorno Moncayo, J.F. Bronchotomy for Metal Foreign Object Removal in Pediatric Airway. J. Pediatr. Surg. Case Rep. 2023, 89, doi:10.1016/j.epsc.2022.102549.
136. Marks, S.C.; Marsh, B.R.; Dudgeon, D.L. Indications for Open Surgical Removal of Airway Foreign Bodies. Ann Otol Rhinol Laryngol 1993, 102, 690–694, doi:10.1177/000348949310200908.
137. Marzabadi, L.R.; Vahdati, S.S.; Tajlil, A. Medical Image. Coughing: Think about Long-Standing Bronchial Foreign Body: Foreign Body Aspiration (FBA). N Z Med J 2012, 125, 110–111.
138. Mathur, N.N.; Ghimire, A.; Joshi, R.R.; Pathak, L. Multiple Factors Responsible for Fatal Outcome in 14-Month-Old Child with 4.5 Cm Right Angled Metallic Nail in the Bronchus. International Journal of Pediatric Otorhinolaryngology Extra 2011, 6, 33–35, doi:10.1016/j.pedex.2010.01.003.
139. Mayr, J.; Dittrich, S.; Triebl, K. A New Method for Removal of Metallic-Ferromagnetic Foreign Bodies from the Tracheobronchial Tree. Pediatric Surgery International 1997, 12, 461–462, doi:10.1007/BF01076970.
140. McAfee, S.J.; Vashisht, R. Removal of an Impacted Distal Airway Foreign Body Using a Guidewire and a Balloon Angioplasty Catheter. Anaesth Intensive Care 2011, 39, 303–304, doi:10.1177/0310057X1103900224.
141. Mehta, D.; Mehta, C.; Bansal, S.; Singla, S.; Tangri, N. Flexible Bronchoscopic Removal of a Three Piece Foreign Body from a Child's Bronchus. Australas Med J 2012, 5, 227–230, doi:10.4066/AMJ.2012.1127.
142. Mekonnen, D. Leech Infestation: The Unusual Cause of Upper Airway Obstruction. Ethiop J Health Sci 2013, 23, 65–68.
143. Mellema, J.D.; Bratton, S.L.; Inglis, A.J.; Morray, J.P. Use of Cardiopulmonary Bypass during Bronchoscopy Following Sand Aspiration. A Case Report. Chest 1995, 108, 1176–1177, doi:10.1378/chest.108.4.1176.
144. Mills, L.J.; Lolley, D.M.; Estrera, A.S.; Platt, M.R. Use of Fluoroscopy in the Removal of Aspirated Foreign Bodies. JAMA: The Journal of the American Medical Association 1977, 237, 1077, doi:10.1001/jama.1977.03270380021004.
145. Montazeri, F.; Bedayat, A.; Jamali, L.; Salehian, M.; Montazeri, G. Leech Endoparasitism: Report of a Case and Review of the Literature. Eur J Pediatr 2009, 168, 39–42, doi:10.1007/s00431-008-0706-1.

146. Morais, I.; Sousa, I.; Martins, A.; Pereira, T.; Costa, V.; Terra, C. Diagnostic Challenges in Paediatric Foreign Body Aspiration: A Case Report. Colombian Journal of Anesthesiology 2021, 49, doi:10.5554/22562087.e919.
147. Morgenstein, K.M. Unlikely Bronchial Foreign Body. Laryngoscope 1970, 80, 467–468, doi:10.1288/00005537-197003000-00009.
148. Morrison, J.B. Inhaled Plastic Cap from Firework: Report of Two Cases. Br Med J 1966, 2, 1052, doi:10.1136/bmj.2.5521.1052.
149. Moskowitz, D.; Gardiner, L.J.; Sasaki, C.T. Foreign-Body Aspiration. Potential Misdiagnosis. Arch Otolaryngol 1982, 108, 806–807, doi:10.1001/archotol.1982.00790600050012.
150. Moss, R.; Kanchanapoon, V. Stone Basket Extraction of a Bronchial Foreign Body. Archives of Surgery 1986, 121, 975, doi:10.1001/archsurg.1986.01400080123027.
151. Ms, V.; Surendran, A.K.; Raja, K. Beyond the Norm: Tracheostomy's Vital Role in Unconventional Foreign Body Removal. Indian J. Otolaryngol. Head Neck Surg. 2023, doi:10.1007/s12070-023-04206-1.
152. Mülazımoğlu, S.; Ocak, E.; Beton, S.; Ozgürsoy, O.B. An Unusual Entry Site for a Nasal Foreign Body: A Neglected Trauma Patient. Ulus Travma Acil Cerrahi Derg 2014, 20, 221–223, doi:10.5505/tjtes.2014.56805.
153. Mundra, R.K.; Agrawal, R.; Sinha, R. Unusual Foreign Body Aspiration in Infants Below 6 Months of Age. Indian Journal of Otolaryngology and Head and Neck Surgery 2014, 66, 145–148, doi:10.1007/s12070-013-0668-0.
154. Munjal, M.; Pooni, P.A.; Sangeeta Endolaryngeal Foreign Bodies. Indian J Pediatr 2000, 67, 847–849, doi:10.1007/BF02726233.
155. Murata, R.; Ozaki, H.; Shiomi, M.; Fujita, K.; Nakagawa, K.; Kaji, A. A Case of Bronchial Cast. Pediatric Radiology 1996, 26, 359–361, doi:10.1007/BF01395716.
156. Nambirajan, L.; Chandrasekharam, V.V.; Bhatnagar, V. Pericardial Foreign Body. J Pediatr Surg 2001, 36, 936–938, doi:10.1053/jpsu.2001.23980.
157. Nanda, N.; Hauser, B.; Heatley, D.; Balasubramaniam, V.; Barreda, C.B. An Unwitnessed Case of Foreign Body Aspiration of Barium from an Unknown Source. Int J Pediatr Otorhinolaryngol 2020, 138, 110355, doi:10.1016/j.ijporl.2020.110355.
158. Nasr, A.; Forte, V.; Friedberg, J.; Langer, J.C. Successful Bronchoscopic Retrieval of Timothy Grass from the Airway. J Pediatr Surg 2005, 40, E39-41, doi:10.1016/j.jpedsurg.2005.01.028.
159. Navalakhe, M.M.; Shah, N.J.; Kirtane, M.V. Foreign Body of Prolonged Duration in the Bronchus--an Unusual Case. Indian J Pediatr 1994, 61, 427–429, doi:10.1007/BF02751908.
160. Newson, T.P.; Parshuram, C.S.; Berkowitz, R.G.; Auldist, A.W.; Robinson, P.J. Tension Pneumothorax Secondary to Grass Head Aspiration. Pediatr Emerg Care 1998, 14, 287–289, doi:10.1097/00006565-199808000-00013.
161. Nozaki, R.; Tsuji, K.; Takebayashi, H.; Hatta, C.; Sakagami, M. A Chopstick as an Epipharyngeal Foreign Body in an Infant. Practica Oto-Rhino-Laryngologica 2003, 96, 1081–1086, doi:10.5631/jibirin.96.1081.
162. Pace-Asciak, P.; Chang, E.; Ludemann, J.P. A Frightening Bronchial Foreign Body. International Journal of Pediatric Otorhinolaryngology Extra 2009, 4, 59–61, doi:10.1016/j.pedex.2008.06.007.

163. Palmer, A.R.; Pollack, M.L. Delayed Diagnosis of Radiopaque Tracheal Foreign Body in a Child. Journal of Emergency Medicine 2006, 31, 427–428, doi:10.1016/j.jemermed.2006.05.022.
164. Parker, K.; Visram, S.; Hodges, S. An Incidental Finding of a Long-Standing Button Battery in the Floor of the Nose during a Routine Orthodontic Examination. J Orthod 2016, 43, 147–150, doi:10.1080/14653125.2016.1158346.
165. Petrovic, S.; Cegar, S.; Lovrenski, J.; Barisic, N.; Till, V. Foreign Body Aspiration in Children-A Diagnostic Challenge. Balkan Medical Journal 2012, 29, 96–98, doi:10.5174/tutfd.2010.04545.1.
166. Philip, J.; Bresnihan, M.; Chambers, N. A Christmas Tree in the Larynx. Paediatr Anaesth 2004, 14, 1016–1020, doi:10.1111/j.1460-9592.2004.01510.x.
167. Poudyal, P.; Tripathi, P.; Guragain, R.P.S.; Rayamajhi, P.; Gyawali, B.R. Natural Expulsion of a Sharp Iron Nail: Right Main Bronchus to the Alimentary Tract: A Case Report. Clinical Case Reports 2021, 9, doi:10.1002/ccr3.4221.
168. Punnoose, S.E.; Victor, J.; Hazarika, P.; Ss, M. C-MAC® Video-Laryngoscope Assisted Removal of Pediatric Upper Airway Foreign Body via Apneic Technique: Two Case Reports. SAGE Open Med Case Rep 2019, 7, 2050313X18823088, doi:10.1177/2050313X18823088.
169. Rahim, S.B.A.; Maruthamuthu, T.; Chooi, L.L.; Singh, A.; Yunus, M.R.B.M. A White Board Needle in the Trachea. Bangladesh Journal of Medical Science 2013, 12, 449–452, doi:10.3329/bjms.v12i4.16668.
170. Rashid, I.; Nichol, P.; Rock, M.J. Aspiration of Grass Head with Subsequent Migration to the Lung Periphery. Pediatric, Allergy, Immunology, and Pulmonology 2015, 28, 191–193, doi:10.1089/ped.2015.0499.
171. Ravikumar, N.; Awasthi, P.; Nallasamy, K.; Angurana, S.K.; Jayashree, M. Impacted Pen Cap in Pharynx Leading to Pharyngojugular and Pharyngocutaneous Fistula in an Infant. J Pediatr Intensive Care 2020, 9, 218–221, doi:10.1055/s-0040-1701208.
172. Razafimanjato, N.N.M.; Ralaivao, R.A.; Ravelomihary, T.D.N.; Hunald, F.A.; Rakotovao, J.L.H. Pneumonectomy in a Child Due to Belated Diagnosis of Foreign Body Aspiration: A Case Report. J Med Case Rep 2021, 15, 533, doi:10.1186/s13256-021-03015-w.
173. Ren, H.; Shi, D.; Gu, Z.; Cao, Z. Simultaneous Esophageal and Tracheal Obstructions Caused by a Pair of Magnetic Beads in a Child: A Case Report. Front Pediatr 2021, 9, 765373, doi:10.3389/fped.2021.765373.
174. Ren, Y.; Zhang, J.; Xin, Z. Anesthetic Management in a 1-Year-Old Child Undergoing Removal of a Large Metal Tracheobronchial Foreign Body. Pediatr Investig 2019, 3, 185–187, doi:10.1002/ped4.12150.
175. Richard, N.; Paygambar, A.; Ducou Le Pointe, H.; Biaz, S.; Corvol, H. Hordeum Murinum Aspiration Revealed by a Pneumopleurocutaneous Fistula in a 15-Month-Old Infant. BMC Pediatr 2021, 21, 550, doi:10.1186/s12887-021-03016-0.
176. Roberts, J.; Bartlett, A.H.; Giannoni, C.M.; Valdez, T.A. Airway Foreign Bodies and Brain Abscesses: Report of Two Cases and Review of the Literature. Int J Pediatr Otorhinolaryngol 2008, 72, 265–269, doi:10.1016/j.ijporl.2007.10.006.

177. Ross, G.L.; Steventon, N.B.; Pinder, D.K.; Bridger, M.W. Living on the Edge of the Post-Nasal Space: The Inhaled Foreign Body. J Laryngol Otol 2000, 114, 56–57, doi:10.1258/0022215001903681.
178. Ross, M.N.; Haase, G.M. An Alternative Approach to Management of Fogarty Catheter Disruption Associated with Endobronchial Foreign Body Extraction. Chest 1988, 94, 882–884, doi:10.1378/chest.94.4.882.
179. Ruangnapa, K.; Anuntaseree, W.; Saelim, K.; Prasertsan, P. Acquired Tracheo-Oesophageal Fistula in a Child with Unrecognised Tracheal Foreign Body. BMJ Case Rep 2021, 14, doi:10.1136/bcr-2020-240947.
180. Ruegemer, J.L.; Perkins, J.A. Combined Rigid and Flexible Endoscopic Removal of a BB Foreign Body from a Peripheral Bronchus. Int J Pediatr Otorhinolaryngol 1999, 47, 77–80, doi:10.1016/s0165-5876(98)00164-5.
181. Sahni, J.K.; Mathur, N.N.; Kansal, Y.; Rana, I. Bronchial Foreign Body Presenting as an Accidental Radiological Finding. Int J Pediatr Otorhinolaryngol 2002, 64, 229–232, doi:10.1016/s0165-5876(02)00002-2.
182. Sakamoto, N.; Fujii, S.; Masumoto, K.; Matsuoka, A.; Toumine, S.; Hara, T.; Shimada, K. Plastic Foreign Body in the Pharynx Can Evade Detection by Computed Tomography. Journal of Pediatric Surgery Case Reports 2022, 84, doi:10.1016/j.epsc.2022.102352.
183. Samdhani, S.; Lohar, N.K.; Singh, A.; Gupta, A. When Bronchoscopy Does Not Suffice for Airway Foreign Body Removal: A Series of Cases Requiring Tracheotomy. Eur. Arch. Oto-Rhino-Laryngol. 2023, 280, 455–459, doi:10.1007/s00405-022-07626-x.
184. Samra, S.; Schroeder, J.W.J.; Valika, T.; Billings, K.R. Tracheotomy for Difficult Airway Foreign Bodies in Children. Otolaryngol Head Neck Surg 2018, 158, 1148–1149, doi:10.1177/0194599818758995.
185. Sarafi M; Rouzrokh M; Sadr S; Mahdavi SA; Mahdavi NS; Ebrahimian M The Usage of a Magnet-Powered Instrument in the Extraction of Metallic or Magnetic Aspirated Foreign Bodies: A Case Report. Int J Surg Case Rep 2022, 99, 107629, doi:10.1016/j.ijscr.2022.107629.
186. Scerbo, C.; Kilgar, J.; Lim, R. Mistaken Asymptomatic Carinal Foreign Body in a Child. Pediatr Emerg Care 2019, 35, e11–e13, doi:10.1097/PEC.0000000000001707.
187. Sedhai, M.; Tripathi, P. Inhaled Open Safety Pin: A Challenging Case. Oxf Med Case Reports 2022, 2022, omac044, doi:10.1093/omcr/omac044.
188. Sembiring, D.; Sibarani, H.; Geary, W.; Han, S. The Cystoscope Used as a Bronchoscope for the Removal of a Foreign Body in the Left Stem Bronchus. Trop Doct 1995, 25, 134, doi:10.1177/004947559502500317.
189. Senthilraj, R.; Amarpreet, S.; Periyathamby, S.; Nik Hassan, N.F.H. It Was Inhaled Not Swallowed-Neglected 5 Month Foreign Body Airway. Bangladesh Journal of Medical Science 2019, 18, 820–822, doi:10.3329/bjms.v18i4.42912.
190. Seth, S.; Kumar, H. An Interesting Case of Button Battery Causing Septal Perforation. Clinical Rhinology 2017, 10, 22–24, doi:10.5005/jp-journals-10013-1298.
191. Shad, R.; Agarwal, A. Broken Safety Pin in Bronchus - Anaesthetic Considerations. Indian Journal of Anaesthesia 2012, 56, 570–571, doi:10.4103/0019-5049.104579.

192. Shamon, S.; Sharma, A.R.; Ludemann, J.P. Glottic Foreign Body in a Child with Pre-Existing Vocal Cord Paralysis and Reflux Laryngitis: A Challenging Diagnostic Paradigm. International Journal of Pediatric Otorhinolaryngology Extra 2011, 6, 122–124, doi:10.1016/j.pedex.2010.05.003.
193. Sharma, A.; Tuteja, S.; Rattan, K.N.; Yadav, R.K. Mummified Insect as Foreign Body in the Respiratory Tract. Indian J Pediatr 1996, 63, 816–818, doi:10.1007/BF02730936.
194. Sharma, H.S.; Sharma, S. Management of Laryngeal Foreign Bodies in Children. J Accid Emerg Med 1999, 16, 150–153, doi:10.1136/emj.16.2.150.
195. Sharma, J.K.; Pippal, S.K.; Sethi, Y.; Arora, S.; Raghuvanshi, S.K. Bronchial Foreign Body: A Case Report. Indian J Otolaryngol Head Neck Surg 2006, 58, 395–396, doi:10.1007/BF03049609.
196. Sharma, P.; Kumar, A. Intraoperative Airway Foreign Body Migration in a Child. Anaesth Intensive Care 2009, 37, 1021–1024, doi:10.1177/0310057X0903700601.
197. Sharma, V.K.; Rana, A.K.; Sharma, R. Uncommon and Dangerous Foreign Body in an Infant's Larynx: An Interesting Presentation. Otorhinolaryngology Clinics 2019, 11, 49–51, doi:10.5005/jp-journals-10003-1313.
198. Shikada, M.; Morikawa, A.; Kobayashi, T.; Nakano, T.; Sakai, T.; Matsuda, S.-I.; Takakura, I.; Shinagawa, T.; Nomura, M.; Niimura, F.; et al. Spontaneous Expectoration of Bronchial Foreign Body: A Case Report. Tokai Journal of Experimental and Clinical Medicine 2007, 32, 83–85.
199. Shivakumar, A.M.; Naik, A.S.; Prashanth, K.B.; Yogesh, B.S.; Shetty, D.K. Unusual Tracheobronchial Foreign Bodies. Indian Journal of Pediatrics 2004, 71, 373–374, doi:10.1007/BF02724114.
200. Shuaib, W.; Khan, M.J.; Alweis, R.; DeMilka, D.Y. A Violent Cough in a 6-Year-Old Boy. Journal of the American Academy of Physician Assistants 2014, 27, 52–53, doi:10.1097/01.JAA.0000455654.42543.44.
201. Sigalet, D.; Lees, G. Tracheoesophageal Injury Secondary to Disc Battery Ingestion. J Pediatr Surg 1988, 23, 996–998, doi:10.1016/s0022-3468(88)80003-4.
202. Simpson, R.; Myer IV, C.; Siracusa, C. Acute Respiratory Distress Syndrome Immediately Following the Removal of an Aspirated Foreign Body. Respiratory Medicine Case Reports 2020, 29, doi:10.1016/j.rmcr.2019.100978.
203. Singh, I.; Gathwala, G.; Yadav, S.P.; Sharma, A. Foreign Body Airway in Neonates. Indian J Pediatr 1999, 66, 288–289, doi:10.1007/BF02761221.
204. Skinner, D.W.; Chui, P. The Hazards Fo "button-Sized" Batteries as Foreign Bodies in the Nose and Ear. Journal of Laryngology and Otology 1986, 100, 1315–1318, doi:10.1017/S0022215100101045.
205. Sobin, L.; Roberson, D.; Watters, K. Use of a Urology Stone Basket for Removal of Subglottic Foreign Bodies. Laryngoscope 2017, 127, 2396–2398, doi:10.1002/lary.26519.
206. Somerville, N.S.; Mearns, C.; Chin, C.; Blaney, S.; Anderson, D. Anesthetic Management of the Complications of Previously Undiagnosed Ingested Foreign Body in a Pediatric Patient. Paediatr Anaesth 2004, 14, 1023–1026, doi:10.1111/j.1460-9592.2004.01312.x.
207. Spencer, M.J.; Millet, V.E.; Dudley, J.P.; Sherrod, J.L.; Bryson, Y.J. Grassheads in the Tracheobronchial Tree: Two Different Outcomes. Ann Otol Rhinol Laryngol 1981, 90, 406–408, doi:10.1177/000348948109000425.

208. Sultan Abdul Kader MI; Syafeera N; Md Nor K; Abu Bakar S; Mat Baki M Supraglottic Foreign Body Missed for One Month in a Child. Cureus 2023, 15, e33870, doi:10.7759/cureus.33870.
209. Sunkum, J.K.A.G. Nasopharyngeal Foreign Body in an Young Child. Indian J Otolaryngol Head Neck Surg 2011, 63, 285–286, doi:10.1007/s12070-011-0191-0.
210. Suryanarayana, K.V.; Chamyal, P.C.; Waghray, M.r. metallic foreign body in left main bronchus (A Case Report). Med J Armed Forces India 1994, 50, 147–148, doi:10.1016/S0377-1237(17)31021-3.
211. Swain, S.K.; Sahu, M.C. An Unusual Presentation of Long Standing Foreign Body at Nasopharynx of a Child – A Case Report. Pediatria Polska 2016, 91, 366–369, doi:10.1016/j.pepo.2016.02.001.
212. Swibel Rosenthal, L.H.; Smith-Bronstein, V.; Cervantes, S.; Schroeder, J.W.J. A Chronic Glottic Foreign Body Diagnosed by Radiograph after 9 Months of Symptoms. Case Rep Pediatr 2018, 2018, 4718428, doi:10.1155/2018/4718428.
213. Szabó, H.; Kálmán, A. Migration of an Aspirated Grass Inflorescence through the Chest Wall in a 3-Year-Old Boy. Pediatric Pulmonology 2021, 56, 344–346, doi:10.1002/ppul.25178.
214. Szczupak, M.; Josephson, G.D. An Unwelcome Party Guest: A Laryngeal Foreign Body Masquerading as Croup. Ear, Nose and Throat Journal 2019, 98, NP147–NP148, doi:10.1177/0145561319878955.
215. Talha, M.A.; Mollah, A.H.; Ahmed, T.; Al Islam, M. A Child with Suppurative Lung Disease by Missed Foreign Body Aspiration: A Case Report. Bangladesh Journal of Medical Science 2021, 20, 208–211, doi:10.3329/bjms.v20i1.50374.
216. Tan, S.S.; Dhara, S.S.; Sim, C.K. Removal of a Laryngeal Foreign Body Using High Frequency Jet Ventilation. Anaesthesia 1991, 46, 741–743, doi:10.1111/j.1365-2044.1991.tb09769.x.
217. Tang, C.L.; Lee, S.C.; Lal, A.B.M.; Thomas, R.A.; Ngui, L.X.; Lim, L.Y. Tracheotomy: An Alternative for Tracheobronchial Foreign Body Removal. Medical Journal of Malaysia 2014, 69, 241–243.
218. Tariq, S.M.; Succony, L.; Bhatia, R.S. Spontaneous Expulsion of a Sharp Foreign Body. J Bronchology Interv Pulmonol 2012, 19, 319–322, doi:10.1097/LBR.0b013e31826c97d1.
219. Taylor, M.A. 2nd; Spanos, S.P.; Fenton, S.J.; Russell, K.W. Ball Magnets Clicked Together on the Epiglottis. Cureus 2020, 12, e8181, doi:10.7759/cureus.8181.
220. Thamboo, A.; Ludemann, J.P.; Riding, K.H. Christmas Decorations May Become Aerodigestive Foreign Bodies. International Journal of Pediatric Otorhinolaryngology Extra 2008, 3, 57–60, doi:10.1016/j.pedex.2007.10.003.
221. Thompson, D.T. Report on a Child with Stricture Following Foreign Body of Long Duration in the Bronchus. Discussion on the Management of Bronchial Foreign Bodies. Postgrad Med J 1979, 55, 886–892, doi:10.1136/pgmj.55.650.886.
222. Thornton, C.S.; Yunker, W.K. Rigid Bronchoscopy and Balloon Dilation for Removal of Aspirated Thumbtacks: Case Series and Literature Review. Int J Pediatr Otorhinolaryngol 2015, 79, 1541–1543, doi:10.1016/j.ijporl.2015.07.007.

223. Tong, C.; Lowinger, D.; Tseros, E.; Wanaguru, D. Inhalation and Ingestion of Magnetic Foreign Bodies in a Toddler with Persistent Cough—a Case Report. Australian Journal of Otolaryngology 2021, doi:10.21037/ajo-20-67.
224. Tsang, J.E.; Sun, J.; Ooi, G.C.; Tsang, K.W. Endobronchial Foreign Body Presenting as Exacerbation of Asthma. Case Rep Emerg Med 2017, 2017, 6863083, doi:10.1155/2017/6863083.
225. Tseng, R.; Stewart, I.; Van Hasselt, A.; Eio, P. Lego Asthma [6]. New England Journal of Medicine 1996, 334, 406–407, doi:10.1056/NEJM199602083340617.
226. Ülk, R.; Onat, S.; Oru, M. Tracheobronchial Pebble Aspiration. Journal of Bronchology 2008, 15, 295–296, doi:10.1097/LBR.0b013e31818953eb.
227. Vahidi, N.A.; Keane, B.A.; Whalen, P.J.; Jeyakumar, A. Hazardous Grilling Due to Wire Brushes. Ear, Nose and Throat Journal 2020, 99, 503–504, doi:10.1177/0145561318824220.
228. Van Dyke, J.J.; Lake, K.B. Survival after Asphyxia Secondary to Gravel Aspiration. Arch Intern Med 1976, 136, 471–473, doi:10.1001/archinte.136.4.471.
229. Vas, L.; Sanzgiri, S.; Patil, B.; Sanghvi, V. An Unusual Cause of Tracheal Stenosis. Can J Anaesth 2000, 47, 261–264, doi:10.1007/BF03018924.
230. Veena, A.; Bist, S.S.; Bharti, B.; Mehrotra, S. Removal of an Aspirated Open Safety Pin from Trachea. Anaesthesia, Pain and Intensive Care 2015, 19, 416–418.
231. Verma, R.K.; Mittal, A.; Kumar, S.; Sahni, J.K. Overlooked Foreign Body at Arytenoid Causing Ulcer on Posterior Pharyngeal Wall: A Case Report and Review of Literature. International Journal of Pediatric Otorhinolaryngology Extra 2012, 7, 55–56, doi:10.1016/j.pedex.2011.10.002.
232. Vlahova A; Antonova Z; Rangelov E; Kartulev N; Oparanova V; Gabrovska N; Spasova A; Velizarova S; Shivachev H Surgical Treatment of Lung Abscess Due to an Awn Aspiration in a 9-Year-Old Child: A Case Report. Children (Basel) 2023, 10, doi:10.3390/children10060910.
233. Walz, P.C.; Scholes, M.A.; Merz, M.N.; Elmaraghy, C.A.; Jatana, K.R. The Internet, Adolescent Males, and Homemade Blowgun Darts: A Recipe for Foreign Body Aspiration. Pediatrics 2013, 132, e519-521, doi:10.1542/peds.2012-3340.
234. Wan Draman, W.N.A.; Ismail, H.; Nik Mohamad, N.K.; Mohamad, I. Intranasal Button Battery: A Case Series. Pediatria i Medycyna Rodzinna 2019, 15, 88–92, doi:10.15557/PiMR.2019.0016.
235. Wang, L.; Zhang, L.; Li, D.; Li, C.; Wang, Y.; Gao, M.; Liang, H.; Meng, F. Successful Retrieval of a Plastic Bead from the Airway of a Child by Flexible Bronchoscopy and a Balloon-Tipped Catheter: A Case Report and Literature Review. Medicine (Baltimore) 2018, 97, e12147, doi:10.1097/MD.0000000000012147.
236. Wankhede, R.G.; Maitra, G.; Pal, S.; Ghoshal, A.; Mitra, S. Successful Removal of Foreign Body Bronchus Using C-Arm-Guided Insertion of Fogarty Catheter through Plastic Bead. Indian J Crit Care Med 2017, 21, 96–98, doi:10.4103/ijccm.IJCCM_148_16.
237. Weston, J.T. Airway Foreign Body Fatalities in Children. Ann Otol Rhinol Laryngol 1965, 74, 1144–1148, doi:10.1177/000348946507400420.
238. Wilkinson, K.A.; Beckett, W.; Brown, T.C.K. Pneumothorax Secondary to Foreign Body Inhalation in a 20 Month Old Child. Journal of Paediatrics and Child Health 1992, 28, 67–68, doi:10.1111/j.1440-1754.1992.tb02621.x.

239. Wineski, R.E.; Panico, E.C.; Bailey, L.N.; Cardenas, A.M.; Grayson, J.W.; Wiatrak, B.J. Flat Sticker as a Mobile Airway Foreign Body: A Case Report and Review of the Literature. Radiology Case Reports 2020, 15, 2391–2395, doi:10.1016/j.radcr.2020.09.031.
240. Wong, K.-S.; Lai, S.-H.; Lien, R.; Hsia, S.-H. Retrieval of Bronchial Foreign Body with Central Lumen Using a Flexible Bronchoscope. International Journal of Pediatric Otorhinolaryngology 2002, 62, 253–256, doi:10.1016/S0165-5876(01)00622-X.
241. Wong, M.; Bhatia, R. A 9-Month-Old with Wheezing and Acute Hypoxic Respiratory Failure. Clin Case Rep 2019, 7, 976–980, doi:10.1002/ccr3.2134.
242. Wu, L.; Sheng, Y.; Xu, X.; Chen, Z.; Wang, Q.; Wang, Z.; Yin, Y. Flexible Bronchoscopy Combined with Rigid Bronchoscopy for Treatment of Scarring in the Bronchus Caused by a Foreign Body. Case Rep Med 2019, 2019, 4616298, doi:10.1155/2019/4616298.
243. Xu, J.; Liu, D.; Huang, Z.; Ke, K. Small Magnet Aspiration as a Pediatric Emergency: A Case Report. Int J Clin Exp Med 2015, 8, 19561–19565.
244. Yang, X.; Ni, X.; Guo, Y.; Zhang, J. Combined Low Tracheotomy and Rigid Bronchoscopy to Remove an Irregular Tracheal Foreign Body. Pediatric Investigation 2018, 2, 196–197, doi:10.1002/ped4.12059.
245. Yeung, J.C.; Smithers, C.J.; Roberson, D.W. Light-Emitting Diode Aspiration: Distinct Radiographic Features and Approach to Management. Int J Pediatr Otorhinolaryngol 2017, 102, 7–9, doi:10.1016/j.ijporl.2017.08.024.
246. Yogev, D.; Bar Moshe, Y.; Tovi, H.; Rekhtman, D. Paroxysmal Upper Airway Obstruction Caused by Two Magnetic Balls. Isr Med Assoc J 2021, 23, 325–326.
247. Zeitlin, J.; Myer, C.M. 3rd Foreign Body in the Trachea. Ann Otol Rhinol Laryngol 2000, 109, 1007–1008, doi:10.1177/000348940010901104.
248. Zhao, Z.G.; Gao, Q.; Song, P.L. A Rare Case of Bilateral Bronchial Foreign Body. Pakistan Journal of Medical Sciences 2015, 31, 477–479, doi:10.12669/pjms.312.6060.

Table S5. List of aspirated foreign bodies

Characteristic	N = 294[1]
Adhesive	3 (1.0%)
Ant	1 (0.3%)
Antenna	1 (0.3%)
Baby wipe	1 (0.3%)
Ball bearing	4 (1.4%)
Ballon	1 (0.3%)
Bark	1 (0.3%)
Bead	9 (3.1%)
Beetle	1 (0.3%)
Bird bones	1 (0.3%)
Blade	6 (2.0%)
Button battery	21 (7.1%)
Button	5 (1.7%)
Can tab	2 (0.7%)
Cap	3 (1.0%)
Candy paper	2 (0.7%)
Cellophane	1 (0.3%)
Chain	3 (1.0%)
Coal piece	1 (0.3%)
Coin	3 (1.0%)
Cotton piece	3 (1.0%)
Dart	5 (1.7%)
Dummy	1 (0.3%)
Elastic	1 (0.3%)
Electronic circuit	1 (0.3%)
Film	1 (0.3%)
Fishing line	1 (0.3%)
Fishing roll bell	1 (0.3%)
Funnel	1 (0.3%)
Glass piece	2 (0.7%)
Grass	9 (3.1%)
Hairpin	1 (0.3%)
Hook	2 (0.7%)
Iron wire	1 (0.3%)
Ketchup envelope	1 (0.3%)
Leaf	2 (0.7%)
Leaves	1 (0.3%)
Led	5 (1.7%)
Leech	4 (1.4%)
Magazine cover piece	1 (0.3%)
Magnets	10 (3.4%)
Metal piece	2 (0.7%)
Nail	11 (3.7%)
Needle	8 (2.7%)
Nettle	1 (0.3%)
Ornamental object	4 (1.4%)
Pacifier	1 (0.3%)

Particles	1 (0.3%)
Pellet	1 (0.3%)
Pen	18 (6.1%)
Pencil	1 (0.3%)
Pin	7 (2.4%)
Plastic piece	6 (2.0%)
Plastic spray cup	1 (0.3%)
Plastic tree piece	2 (0.7%)
Plastic wrapper	1 (0.3%)
Plat piece	2 (0.7%)
Ring	4 (1.4%)
Safety pin	10 (3.4%)
Sand	4 (1.4%)
Scarp piece	1 (0.3%)
Screw	9 (3.1%)
Seed (spiny cocklebus)	1 (0.3%)
Snack wrapping	1 (0.3%)
Spike	1 (0.3%)
Spike (Hordenum marinum)	2 (0.7%)
Splinter	1 (0.3%)
Sponge	1 (0.3%)
Spring	6 (2.0%)
Sticker	2 (0.7%)
Stone	6 (2.0%)
Tablet	1 (0.3%)
Tablet package	1 (0.3%)
Thumbtack	8 (2.7%)
Toothbrush cover	3 (1.0%)
Toy	20 (6.8%)
Tube	5 (1.7%)
Twig	2 (0.7%)
Wall plug	1 (0.3%)
Wand	3 (1.0%)
Wedge	1 (0.3%)
Whistle	6 (2.0%)
Wire	3 (1.0%)

Table S6. Joanna Briggs Institute (JBI) checklist score of the case reports included in the review

Author year	Q1	Q2	Q3	Q4	Q5	Q6	Q7	Q8
Abbas 2022	Yes	No	Yes	Yes	Yes	Yes	No	Yes
Abraham 2020	No	No	Yes	Yes	No	Yes	Yes	Yes
Adoga 2009	Yes	No	Yes	Yes	No	Yes	No	Yes
Ahad 1999	No	No	Yes	Yes	No	No	Yes	Yes
Aihole 2019	No	Yes	Yes	Yes	No	No	Yes	Yes
Aihole 2020	No	No	Yes	Yes	No	No	No	Yes
Akhter 1994	Yes	No	Yes	Yes	No	No	Yes	Yes
Aljahdali 2021	Yes	Yes	Yes	Yes	Yes	Yes	Yes	Yes
AlKhalifah 2022	Yes	No	Yes	Yes	Yes	Yes	Yes	Yes
Alotaibi 2022	No	Yes	Yes	Yes	Yes	No	No	Yes
Ambu 2001	Yes	Yes	Yes	Yes	Yes	Yes	Yes	Yes
Anajar 2017	Yes	Yes	Yes	Yes	Yes	Yes	Yes	Yes
Anand 1979	Yes	No	Yes	Yes	Yes	No	No	Yes
Antón-Pacheco 2008	Yes	Yes	Yes	Yes	Yes	Yes	Yes	Yes
ArunBabu 2013	Yes	No	Yes	Yes	Yes	No	No	Yes
Arutyunyan 2014	Yes	No	Yes	Yes	Yes	Yes	No	Yes
Asaf 2017	Yes	No	Yes	Yes	Yes	Yes	Yes	Yes
Atmaca 2009	Yes	Yes	Yes	Yes	Yes	No	No	Yes
Atmaca 2011	Yes	No	Yes	No	Yes	No	No	No
Azurara 2016	Yes	Yes	Yes	Yes	Yes	No	No	Yes
Baker 1989	Yes	Yes	Yes	Yes	Yes	Yes	Yes	Yes
Bakhshaee 2012	Yes	Yes	Yes	Yes	Yes	Yes	Yes	Yes
Bakshi 2007	Yes	Yes	Yes	Yes	Yes	Yes	Yes	Yes
Bakshi 2016	Yes	Yes	Yes	Yes	Yes	Yes	Yes	Yes
Baliarsingh 2017	Yes	Yes	Yes	Yes	Yes	Yes	Yes	Yes
Barrett 1995	Yes	Yes	Yes	Yes	Yes	Yes	Yes	Yes
Başok 1997	Yes	Yes	Yes	Yes	Yes	Yes	Yes	Yes
Bhat 1996	Yes	Yes	Yes	Yes	Yes	Yes	Yes	Yes
Boleken 2005	Yes	Yes	Yes	Yes	Yes	Yes	Yes	Yes
Bradshaw 2019	Yes	Yes	Yes	Yes	Yes	Yes	Yes	Yes
Brand 2003	Yes	Yes	Yes	Yes	Yes	Yes	Yes	Yes
Brown 1994	Yes	No	Yes	No	No	Yes	No	Yes
Bukhari 2022	Yes	No	Yes	Yes	Yes	Yes	No	Yes
Cakir 2012	Yes	Yes	Yes	Yes	Yes	Yes	Yes	Yes
Capo 1986	Yes	Yes	Yes	Yes	No	Yes	Yes	Yes
Chang 2015	Yes	Yes	No	No	Yes	Yes	No	Yes
Chaudhry 2020	Yes	Yes	Yes	Yes	Yes	Yes	Yes	Yes
Chen 2018	Yes	Yes	Yes	Yes	Yes	Yes	Yes	Yes
Chhangani 1966	Yes	Yes	Yes	Yes	Yes	Yes	Yes	Yes
Choy 1996	Yes	Yes	Yes	Yes	Yes	Yes	Yes	Yes
Chua 2006	Yes	Yes	Yes	Yes	No	Yes	Yes	Yes
Cleveland 1998	Yes	Yes	Yes	Yes	Yes	Yes	Yes	Yes
Concerto 2018	Yes	No	Yes	Yes	Yes	No	No	No
Daines 2008	Yes	Yes	Yes	Yes	Yes	Yes	No	Yes

Datema 2009	Yes	Yes	Yes	Yes	Yes	Yes	Yes	Yes
Dave 2007	No	No	Yes	Yes	Yes	No	Yes	Yes
Davis 2007	Yes	Yes	Yes	Yes	Yes	Yes	Yes	Yes
Davis 2019	Yes	Yes	Yes	Yes	Yes	Yes	Yes	Yes
DeJesusBarbosa 2016	Yes	No	Yes	Yes	Yes	Yes	No	Yes
Deng 2010	Yes	Yes	Yes	Yes	Yes	Yes	Yes	Yes
Deng 2016	Yes	No	Yes	Yes	Yes	Yes	No	No
Dinleyici 2008	Yes	Yes	Yes	Yes	Yes	Yes	No	Yes
Dorfman 2011	Yes	Yes	Yes	Yes	Yes	Yes	Yes	Yes
Doyle 2009	Yes	No	Yes	No	Yes	No	No	Yes
Eghtedari 2003	Yes	No	Yes	Yes	No	No	No	Yes
Eun 1984	Yes	Yes	Yes	Yes	Yes	Yes	No	Yes
Fang 2016	Yes	Yes	Yes	Yes	Yes	Yes	Yes	Yes
Feng 2016	Yes	Yes	Yes	Yes	Yes	Yes	Yes	Yes
Findlay 2003	Yes	No	Yes	Yes	Yes	Yes	No	Yes
Fosarelli 1988	Yes	Yes	Yes	Yes	Yes	Yes	Yes	Yes
Fraccaroli 2023	Yes	Yes	Yes	Yes	Yes	Yes	No	No
Fraga 2002	Yes	Yes	Yes	Yes	Yes	Yes	Yes	Yes
Freiman 2001	Yes	Yes	Yes	Yes	Yes	Yes	Yes	Yes
Garg 2010	Yes	Yes	Yes	Yes	Yes	No	Yes	Yes
Gerber 2020	Yes	Yes	Yes	Yes	Yes	Yes	No	Yes
GibsonJr 2000	Yes	No	Yes	Yes	Yes	Yes	No	Yes
Gilbert 1970	Yes	Yes	Yes	Yes	Yes	No	No	No
Goez 1994	Yes	Yes	Yes	Yes	Yes	Yes	No	No
Goh 2015	Yes	Yes	Yes	Yes	Yes	Yes	Yes	Yes
Gomez-Acevedo 2010	Yes	Yes	Yes	Yes	Yes	Yes	Yes	Yes
Gómez-Ramos 2022	Yes	Yes	Yes	Yes	Yes	Yes	Yes	Yes
Goswami 2016	Yes	Yes	Yes	Yes	Yes	Yes	Yes	Yes
Goussard 2020	Yes	Yes	Yes	Yes	Yes	Yes	Yes	Yes
Goussard 2021	Yes	Yes	Yes	Yes	Yes	Yes	Yes	Yes
Goyal 2007	Yes	Yes	Yes	Yes	Yes	Yes	Yes	Yes
Gupta 2010	Yes	Yes	Yes	Yes	Yes	Yes	Yes	Yes
Hada 2012	Yes	Yes	Yes	Yes	Yes	Yes	Yes	Yes
Hainer 2016	Yes	Yes	Yes	Yes	Yes	Yes	Yes	Yes
Haloob 2014	Yes	Yes	Yes	Yes	Yes	Yes	Yes	Yes
Hamidi 2022	Yes	Yes	Yes	Yes	Yes	Yes	Yes	Yes
Hemead 2021	Yes	Yes	Yes	Yes	No	Yes	Yes	Yes
Heyworth 2019	Yes	Yes	No	No	No	No	No	No
Hickey 2022	Yes	Yes	Yes	Yes	Yes	Yes	Yes	Yes
Hiebert 2016	Yes	Yes	No	No	No	No	No	Yes
Hilman 1980	Yes	Yes	Yes	Yes	Yes	Yes	Yes	Yes
Hootnick 2015	Yes	Yes	Yes	Yes	Yes	Yes	Yes	Yes
Hosokawa 2022	Yes	Yes	Yes	No	Yes	Yes	No	Yes
Hussain 1994	Yes	Yes	Yes	Yes	Yes	Yes	Yes	Yes
Idris 2018	Yes	Yes	Yes	Yes	Yes	No	Yes	Yes
Iqbal 2011	Yes	Yes	Yes	Yes	Yes	Yes	Yes	Yes
Issaka 2023	Yes	No	Yes	Yes	Yes	Yes	No	Yes
Jabbardarjani 2009	Yes	Yes	Yes	Yes	Yes	Yes	Yes	Yes
Jain 2013	Yes	Yes	Yes	Yes	Yes	Yes	Yes	Yes

Jardeleza 2021	Yes	Yes	Yes	Yes	Yes	Yes	Yes	Yes
Jayaraj 2017	Yes	No	Yes	Yes	Yes	No	No	Yes
Jean 2022	Yes	No	Yes	Yes	Yes	No	No	Yes
Jotdar 2015	Yes	No	Yes	Yes	Yes	Yes	Yes	Yes
Jotdar 2016	Yes	No	Yes	Yes	Yes	Yes	No	Yes
Kansal 2015	Yes	No	Yes	No	No	No	No	No
Kara 2022	Yes	Yes	Yes	Yes	Yes	Yes	Yes	Yes
Kasuka 2022	Yes	Yes	Yes	Yes	Yes	Yes	Yes	Yes
Kathuria 2017	Yes	Yes	Yes	Yes	Yes	Yes	Yes	Yes
Katz 1979	Yes	Yes	Yes	No	No	Yes	No	Yes
Kawano 2007	Yes	Yes	Yes	Yes	Yes	Yes	Yes	Yes
Kazi 2015	Yes	Yes	Yes	Yes	Yes	Yes	Yes	Yes
Kent 1990	Yes	Yes	Yes	Yes	Yes	Yes	Yes	Yes
Kim 2021	Yes	Yes	Yes	Yes	Yes	Yes	Yes	Yes
Kondo 2006	Yes	Yes	Yes	Yes	Yes	Yes	Yes	Yes
Kumar 2003	Yes	Yes	Yes	Yes	Yes	Yes	Yes	Yes
Kumar 2013	Yes	Yes	Yes	Yes	Yes	Yes	Yes	Yes
Kumar 2013	Yes	Yes	Yes	Yes	Yes	Yes	Yes	Yes
Kumar 2013	Yes	Yes	Yes	Yes	Yes	Yes	Yes	Yes
Kurul 2002	Yes	Yes	Yes	Yes	Yes	No	No	Yes
Lau 2015	Yes	Yes	Yes	Yes	Yes	Yes	Yes	Yes
Lavarde 2003	Yes	Yes	Yes	Yes	Yes	Yes	Yes	Yes
Leffler 2006	Yes	Yes	Yes	Yes	Yes	Yes	Yes	Yes
Leiberman 1985	Yes	Yes	Yes	Yes	Yes	Yes	Yes	Yes
Leonard 2015	Yes	Yes	Yes	Yes	Yes	Yes	Yes	Yes
Lima 2017	Yes	Yes	Yes	Yes	Yes	Yes	Yes	Yes
Liman 2012	Yes	No	No	No	No	No	No	Yes
Little 2000	Yes	Yes	Yes	Yes	Yes	Yes	Yes	Yes
Lloyd-Thomas 1986	Yes	Yes	Yes	Yes	Yes	Yes	Yes	Yes
Łoś-Rycharska 2021	Yes	Yes	Yes	Yes	Yes	Yes	Yes	Yes
Lukse 2014	Yes	Yes	Yes	Yes	Yes	Yes	Yes	Yes
MacNeil 2010	Yes	Yes	Yes	Yes	Yes	Yes	Yes	Yes
Maglione 2022	Yes	Yes	Yes	Yes	Yes	Yes	No	No
Maguire 2012	Yes	Yes	Yes	Yes	Yes	Yes	Yes	Yes
Majd 1977	Yes	Yes	Yes	Yes	Yes	Yes	Yes	Yes
MarínGiraldo 2023	Yes	Yes	Yes	Yes	Yes	Yes	Yes	Yes
Marzabadi 2012	Yes	Yes	Yes	Yes	Yes	No	No	No
Mathur 2011	Yes	Yes	Yes	Yes	Yes	Yes	Yes	Yes
Mayr 1997	Yes	Yes	Yes	Yes	Yes	Yes	Yes	Yes
McAfee 2011	Yes	Yes	Yes	Yes	Yes	Yes	Yes	Yes
Mehta 2012	Yes	Yes	Yes	Yes	Yes	Yes	Yes	Yes
Mekonnen 2013	Yes	Yes	Yes	Yes	Yes	Yes	Yes	Yes
Mellema 1995	Yes	Yes	Yes	Yes	Yes	Yes	Yes	Yes
Montazeri 2009	Yes	Yes	Yes	Yes	Yes	Yes	Yes	Yes
Morais 2021	Yes	Yes	Yes	Yes	Yes	Yes	Yes	Yes
Morgenstein 1970	Yes	Yes	Yes	Yes	Yes	No	No	No
Moskowitz 1982	Yes	Yes	Yes	Yes	Yes	Yes	Yes	Yes
Moss 1986	Yes	Yes	Yes	No	Yes	No	No	No
Ms 2023	Yes	Yes	Yes	Yes	Yes	Yes	Yes	Yes

Mülazımoğlu 2014	Yes	Yes	Yes	Yes	Yes	Yes	Yes	Yes
Munjal 2000	No	No	Yes	Yes	Yes	No	No	Yes
Murata 1996	Yes	Yes	Yes	Yes	Yes	No	Yes	Yes
Nambirajan 2001	Yes	Yes	Yes	Yes	Yes	Yes	Yes	Yes
Nanda 2020	Yes	Yes	Yes	Yes	Yes	Yes	Yes	Yes
Navalakhe 1994	Yes	Yes	Yes	Yes	Yes	Yes	Yes	Yes
Newson 1998	Yes	Yes	Yes	Yes	Yes	Yes	Yes	Yes
Nozaki 2003	Yes	Yes	Yes	Yes	Yes	Yes	Yes	Yes
Pace-Asciak 2009	Yes	Yes	Yes	Yes	Yes	Yes	Yes	Yes
Palmer 2006	Yes	Yes	Yes	Yes	Yes	Yes	Yes	Yes
Parker 2016	Yes	Yes	Yes	Yes	Yes	Yes	Yes	Yes
Petrovic 2012	Yes	Yes	Yes	Yes	Yes	Yes	Yes	Yes
Philip 2004	Yes	Yes	Yes	Yes	Yes	Yes	Yes	Yes
Poudyal 2021	Yes	Yes	Yes	Yes	Yes	Yes	Yes	Yes
Punnoose 2019	Yes	Yes	Yes	Yes	Yes	Yes	Yes	Yes
Rahim 2013	Yes	Yes	Yes	Yes	Yes	Yes	Yes	Yes
Rashid 2015	Yes	Yes	Yes	Yes	Yes	Yes	Yes	Yes
Ravikumar 2020	Yes	Yes	Yes	Yes	Yes	Yes	Yes	Yes
Razafimanjato 2021	Yes	Yes	Yes	Yes	Yes	Yes	Yes	Yes
Ren 2019	Yes	Yes	Yes	Yes	Yes	Yes	Yes	Yes
Ren 2021	Yes	Yes	Yes	Yes	Yes	Yes	Yes	Yes
Richard 2021	Yes	Yes	Yes	Yes	Yes	Yes	Yes	Yes
Roberts 2008	Yes	Yes	Yes	Yes	Yes	Yes	Yes	Yes
Ross 1988	Yes	Yes	Yes	Yes	Yes	Yes	Yes	Yes
Ross 2000	Yes	Yes	Yes	Yes	Yes	Yes	Yes	Yes
Ruangnapa 2021	Yes	Yes	Yes	Yes	Yes	Yes	Yes	Yes
Ruegemer 1999	Yes	Yes	Yes	Yes	Yes	Yes	Yes	Yes
Sahni 2002	Yes	Yes	Yes	Yes	Yes	Yes	Yes	Yes
Sakamoto 2022	Yes	Yes	Yes	Yes	Yes	Yes	Yes	Yes
Samra 2018	Yes	Yes	Yes	Yes	Yes	Yes	Yes	Yes
Sarafi 2022	Yes	Yes	Yes	Yes	Yes	Yes	Yes	Yes
Scerbo 2019	Yes	Yes	Yes	Yes	Yes	Yes	Yes	Yes
Sedhai 2022	Yes	Yes	Yes	Yes	Yes	Yes	Yes	Yes
Sembiring 1995	Yes	Yes	No	No	Yes	No	No	No
Senthilraj 2019	Yes	Yes	Yes	Yes	Yes	Yes	Yes	Yes
Seth 2017	Yes	Yes	Yes	Yes	Yes	Yes	Yes	Yes
Shad 2012	Yes	Yes	Yes	Yes	Yes	Yes	Yes	Yes
Shamon 2011	Yes	Yes	Yes	Yes	Yes	Yes	Yes	Yes
Sharma 1996	Yes	Yes	Yes	Yes	Yes	Yes	Yes	Yes
Sharma 1999	Yes	Yes	Yes	Yes	Yes	Yes	Yes	Yes
Sharma 2006	Yes	Yes	Yes	Yes	Yes	Yes	Yes	Yes
Sharma 2009	Yes	Yes	Yes	Yes	Yes	Yes	Yes	Yes
Sharma 2019	Yes	Yes	Yes	Yes	Yes	Yes	Yes	Yes
Shikada 2007	Yes	Yes	Yes	Yes	Yes	Yes	Yes	Yes
Shivakumar 2004	Yes	Yes	Yes	Yes	Yes	Yes	Yes	Yes
Shuaib 2014	Yes	Yes	Yes	Yes	Yes	Yes	No	No
Sigalet 1988	Yes	Yes	Yes	Yes	Yes	Yes	Yes	Yes
Simpson 2020	Yes	Yes	Yes	Yes	Yes	Yes	Yes	Yes
Singh 1999	Yes	Yes	Yes	Yes	Yes	No	No	Yes

Skinner 1986	Yes	Yes	Yes	Yes	Yes	Yes	Yes	Yes
Sobin 2017	Yes	Yes	Yes	Yes	Yes	Yes	Yes	Yes
Somerville 2004	Yes	Yes	Yes	Yes	Yes	Yes	Yes	Yes
Spencer 1981	Yes	Yes	Yes	Yes	Yes	Yes	Yes	Yes
SultanAbdulKader 2023	Yes	Yes	Yes	Yes	Yes	Yes	Yes	Yes
Sunkum 2011	Yes	Yes	Yes	Yes	Yes	Yes	Yes	Yes
Suryanarayana 1994	Yes	Yes	Yes	Yes	Yes	Yes	Yes	Yes
Swain 2016	Yes	Yes	Yes	Yes	Yes	Yes	Yes	Yes
SwibelRosenthal 2018	Yes	Yes	Yes	Yes	Yes	Yes	Yes	Yes
Szabó 2021	Yes	Yes	Yes	Yes	Yes	Yes	Yes	Yes
Szczupak 2019	Yes	Yes	Yes	Yes	Yes	Yes	No	No
Talha 2021	Yes	Yes	Yes	Yes	Yes	Yes	Yes	Yes
Tan 1991	Yes	Yes	Yes	Yes	Yes	Yes	Yes	Yes
Tang 2014	Yes	Yes	Yes	Yes	Yes	Yes	Yes	Yes
Tariq 2012	Yes	Yes	Yes	Yes	Yes	Yes	Yes	Yes
Taylor 2020	Yes	Yes	Yes	Yes	Yes	Yes	Yes	Yes
Thamboo 2008	Yes	Yes	Yes	Yes	Yes	Yes	No	Yes
Thompson 1979	Yes	Yes	Yes	Yes	Yes	Yes	Yes	Yes
Thornton 2015	Yes	Yes	Yes	Yes	Yes	Yes	Yes	Yes
Tsang 2017	Yes	Yes	Yes	Yes	Yes	Yes	Yes	Yes
Tseng 1996	Yes	Yes	Yes	Yes	Yes	Yes	Yes	Yes
Ülk 2008	Yes	Yes	Yes	Yes	Yes	Yes	Yes	Yes
Vahidi 2020	Yes	Yes	Yes	Yes	Yes	Yes	Yes	Yes
VanDyke 1976	Yes	Yes	Yes	Yes	Yes	Yes	Yes	Yes
Vas 2000	Yes	Yes	Yes	Yes	Yes	Yes	Yes	Yes
Veena 2015	Yes	Yes	Yes	Yes	Yes	Yes	Yes	Yes
Verma 2012	Yes	Yes	Yes	Yes	Yes	Yes	Yes	Yes
Vlahova 2023	Yes	Yes	Yes	Yes	Yes	Yes	Yes	Yes
Walz 2013	Yes	Yes	Yes	Yes	Yes	Yes	Yes	Yes
WanDraman 2019	Yes	Yes	Yes	Yes	Yes	Yes	Yes	Yes
Wang 2018	Yes	No	Yes	Yes	Yes	No	No	Yes
Wankhede 2017	Yes	Yes	Yes	Yes	Yes	Yes	Yes	Yes
Wilkinson 1992	Yes	Yes	Yes	Yes	Yes	Yes	No	Yes
Wineski 2020	Yes	Yes	Yes	Yes	Yes	Yes	Yes	Yes
Wong 2002	Yes	Yes	Yes	Yes	Yes	Yes	Yes	Yes
Wong 2019	Yes	Yes	Yes	Yes	Yes	Yes	Yes	Yes
Wu 2019	Yes	Yes	Yes	Yes	Yes	Yes	Yes	Yes
Xu 2015	Yes	Yes	Yes	Yes	Yes	Yes	Yes	Yes
Yang 2018	Yes	Yes	Yes	Yes	Yes	Yes	Yes	Yes
Yeung 2017	Yes	Yes	Yes	Yes	Yes	Yes	Yes	Yes
Yogev 2021	Yes	Yes	Yes	Yes	Yes	Yes	Yes	Yes
Zeitlin 2000	Yes	Yes	Yes	Yes	Yes	Yes	Yes	Yes
Zhao 2015	Yes	Yes	Yes	Yes	Yes	Yes	Yes	Yes

Y = Yes; N = No; U = Unclear; NA = Not Applicable.

Questions from the JBI Checklist

Q1 Were patient's demographic characteristics clearly described?

Q2 Was the patient's history clearly described and presented as a timeline?

Q3 Was the current clinical condition of the patient on presentation described in detail?
Q4 Were diagnostics tests or assessment methods and the results clearly described?
Q5 Was the intervention(s) or treatment procedure(s) clearly described?
Q6 Was the post-intervention clinical condition clearly described?
Q7 Were adverse events (harms) or unanticipated events identified and described?
Q8 Does the case report provide take-away lessons?

Table S7. Joanna Briggs Institute (JBI) checklist score of the case series included in the review

Author year	Q1	Q2	Q3	Q4	Q5	Q6	Q7	Q8	Q9	Q10
Al-Halfawy 2007	Yes	Yes	Yes	Yes	Yes	No	Yes	No	No	No
Humphries 1988	Yes	No	No	No	Yes	No	Yes	No	No	No
Ludemann 2007	Yes	No	Yes	Yes	Yes	No	Yes	Yes	No	No
Liao 2015	Yes	Yes	Yes	Yes	Yes	Yes	Yes	Yes	Yes	Yes
Weston 1965	Yes	Yes	Yes	Yes	Yes	Yes	Yes	Yes	Yes	No
Samdhani 2023	Yes	Yes	Yes	Yes	Yes	Yes	Yes	Yes	Yes	Yes
Nasr 2005	Yes	Yes	Yes	Yes	Yes	Yes	Yes	Yes	Yes	Yes
Marks 1993	Yes	Yes	Yes	Yes	Yes	Yes	Yes	Yes	Yes	Yes
Abder-Rahman 2009	Yes	Yes	Yes	Yes	Yes	Yes	Yes	Yes	Yes	Yes
Morrison 1966	Yes	Yes	Yes	Yes	Yes	Yes	Yes	Yes	Yes	Yes
Mills 1977	Yes	Yes	Yes	Yes	Yes	No	No	No	No	No
Mundra 2014	Yes	Yes	Yes	Yes	Yes	Yes	Yes	Yes	Yes	Yes
Tong 2021	Yes	Yes	Yes	Yes	Yes	Yes	Yes	Yes	Yes	Yes
Loh 2003	No	Yes	Yes	No	Yes	Yes	Yes	Yes	Yes	No

Y = Yes; N = No; U = Unclear; NA = Not Applicable.

Questions from the JBI Checklist

Q1 Were there clear criteria for inclusion in the case series?

Q2 Was the condition measured in a standard, reliable way for all participants included in the case series?

Q3 Were valid methods used for identification of the condition for all participants included in the case series?

Q4 Did the case series have consecutive inclusion of participants?

Q5 Did the case series have complete inclusion of participants?

Q6 Was there clear reporting of the demographics of the participants included in the study?

Q7 Was there clear reporting of clinical information of the participants?

Q8 Were the outcomes or follow-up results of cases clearly reported?

Q9 Was there clear reporting of the presenting sites'/clinics' demographic information?

Q10 Was statistical analysis appropriate?
